# Organo‐Hydrogel Electrolytes with Versatile Environmental Adaptation for Advanced Flexible Aqueous Energy Storage Devices

**DOI:** 10.1002/smsc.202200104

**Published:** 2023-04-05

**Authors:** Hongfei Wang, Muhammad Sohail Riaz, Tariq Ali, Jiawei Gu, Yijun Zhong, Yong Hu

**Affiliations:** ^1^ Key Laboratory of the Ministry of Education for Advanced Catalysis Materials Department of Chemistry Zhejiang Normal University Jinhua 321004 China; ^2^ Hangzhou Institute of Advanced Studies Zhejiang Normal University Hangzhou 311231 China

**Keywords:** anti-freezing capability, environmental adaptation, flexible devices, mechanical strength, organo-hydrogel electrolytes

## Abstract

With growing demands for portable electronics, flexible aqueous energy storage devices such as supercapacitors and Zn‐based batteries have drawn tremendous interest from both academic and industrial fields. Organo‐hydrogels, crosslinked amphiphilic polymers filled with organic solvents and water, are regarded as an ideal electrolyte candidate for portable electronic applications, especially due to their superior environmental adaptation. Organo‐hydrogels can reserve the advantages of the corresponding hydrogels and achieve some unique features such as broad temperature tolerance, high mechanical stability, strong interfacial interaction, and decent ionic conductivity. This review outlines the composition fundamentals, preparation methods, and comprehensive properties of reported organo‐hydrogel electrolytes. In particular, supercapacitors and Zn‐based batteries that operate under harsh temperature conditions and unusual mechanical deformations endowed by organo‐hydrogel electrolytes are further highlighted. Moreover, a unique perspective on the current challenges and future development directions of the organo‐hydrogel electrolytes for flexible energy storage is also provided.

## Graphical abstract

This review systematically summarizes the recent advances of organo‐hydrogel electrolytes with versatile environmental adaptation in flexible aqueous energy storage devices, including supercapacitors, Zn‐ion batteries, and Zn–air batteries. Moreover, several critical scientific challenges and research perspectives about organo‐hydrogel electrolytes are also discussed.

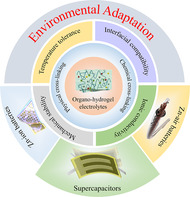

## 1 Introduction

Recently, wearable electronics with unique ductility, comfortability, and low‐cost manufacturing process have sparked extensive applications in information engineering, energy storage/conversion, medical instruments, and national defense.^[^ 1, 2, 3 ^]^ To satisfy the particular requirements of these devices, flexible power sources must be developed that can withstand variable mechanical strains and harsh environmental conditions.^[^ 4, 5 ^]^ Considering this perspective, stretchable gel materials with unique polymer network structures are perceived as promising candidates, which can render the components in energy storage devices with recoverable shapes and high flexibility.^[^ 6, 7 ^]^

Dispersing polymer chains in different solvent media is conducive to preparing gel materials with desired properties, such as hydrogels,^[^ 7, 8, 9 ^]^ organogels,^[^ 10, 11 ^]^ and ionogels.^[^ 12, 13, 14 ^]^ In particular, hydrogels typically consist of 3D elastic crosslinked polymer networks with interstitials filled with a large amount of water, delivering both liquid‐like transport capacity and solid‐like mechanical strength.^[^ 9, 15 ^]^ Nowadays, many reviews have reported the application of hydrogels in electrochemistry.^[^ 8, 9 ^]^ Nevertheless, the practical use of hydrogels still encounters many bottleneck issues. Due to the high content of water, the issues of freezing hardening at subzero temperatures and evaporation‐induced structural dehydration in dry environments will inevitably reside in hydrogels, resulting in the loss of their multiple functionalities eventually.^[^ ^16^ ^]^ Besides, the balance between high energy density and additional functions (self‐healing, scalability, deformation, adhesiveness, and so on) is rarely accomplished in traditional hydrogels.^[^ ^17^ ^]^

It is of practical significance to develop gel materials with wide environmental adaptability and promising energy storage application prospects.^[^ ^18^ ^]^ In contrast, it is quite desirable to construct organo‐gel/hydrogel hybrids based on opposite physicochemical properties to attain a wealth of distinctive functions.^[^ ^19^ ^]^ Correspondingly, incorporating organic solvents into hydrogels has developed various organo‐hydrogels with superior resistance against freezing or drying to eliminate electro‐mechanical performance deterioration under extreme environmental conditions.^[^ 20, 21, 22 ^]^ Therefore, organo‐hydrogels are considered ideal innovative materials to suit flexible energy storage applications once flexibility, temperature adaptation, and ionic conductivity are perfectly combined.

Electrolytes bridging two electrodes are one of the most crucial components in flexible energy storage/conversion devices since they are directly related to ionic transportation, electrochemical stable potential window (ESPW), and cycling stability.^[^ 23, 24 ^]^ To accomplish the flexibility of devices, good compatibility between soft electrolytes and other components is crucial.^[^ ^25^ ^]^ Among multitudinous flexible electrolytes, organo‐hydrogel electrolytes are usually composed of polymer networks, mixed solvents, and conductive salts, which are primarily fostered in response to the shortcomings of traditional liquid electrolytes and pure hydrogel electrolytes.^[^ ^26^ ^]^ Particularly, organo‐hydrogel electrolytes generally display better assembly integrity than liquid electrolytes, and more stable environmental adaptability than hydrogel electrolytes, and no additional separators are required as they could both serve as separators and electrolytes.^[^ ^27^ ^]^ The binary synergy principle is subtly applied to design hydrophilic/oleophilic complemental polymeric gel materials, where organic solvents as protectants cooperate with water to provide a medium for ions transport. Furthermore, the modified solvent environment in organo‐hydrogel electrolytes also allows for broadening the application of flexible aqueous energy storage devices, such as supercapacitors^[^ 28, 29, 30 ^]^ and Zn‐based batteries.^[^ ^31^ ^]^ Over the years, numerous efforts have been dedicated to developing soft organo‐hydrogel electrolytes to overcome the challenges in practice: 1) preservation of ionic conductivity at extremely low temperatures (below –20 °C);^[^ ^32^ ^]^ 2) avoidance of dehydration, salt crystallization and thermal shock at extremely high temperatures (over 50 °C);^[^ ^33^ ^]^ 3) firm adhesiveness at repeated deformations.^[^ ^34^ ^]^

**Scheme 1:**
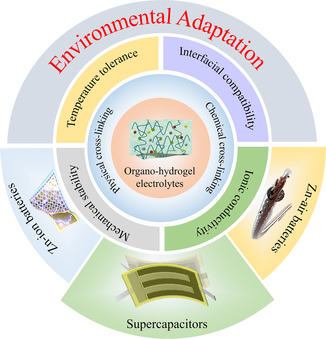
General synthesis strategies and unique properties of organo‐hydrogel electrolytes, and their applications for advanced flexible aqueous energy storage devices.

To the best of our knowledge, there has been no systematic overview focusing on organo‐hydrogel electrolytes for flexible aqueous energy storage devices. To obtain an in‐depth understanding of the recent progress and specify the representative achievements, it is essential to make a comprehensive summary of organo‐hydrogel electrolytes aiming at wide environmental adaptation and excellent electrochemical performance in this field (**Scheme** **1**). This review first covers various methods to optimize the physicochemical properties of organo‐hydrogels in terms of temperature tolerance, mechanical stability, interfacial compatibility, and ionic conductivity by adding inorganic/organic solvents. Subsequently, the studies of various organo‐hydrogel electrolytes with enriched intelligent functionalities, such as stretchability, self‐healing, anti‐freezing, and thermotolerance in response to external environment change, are particularly highlighted. Finally, the existing challenges and relevant perspectives are further proposed to deepen the research in this field.

## 2 Fundamentals of Organo‐Hydrogel Electrolytes

**Figure 1:**
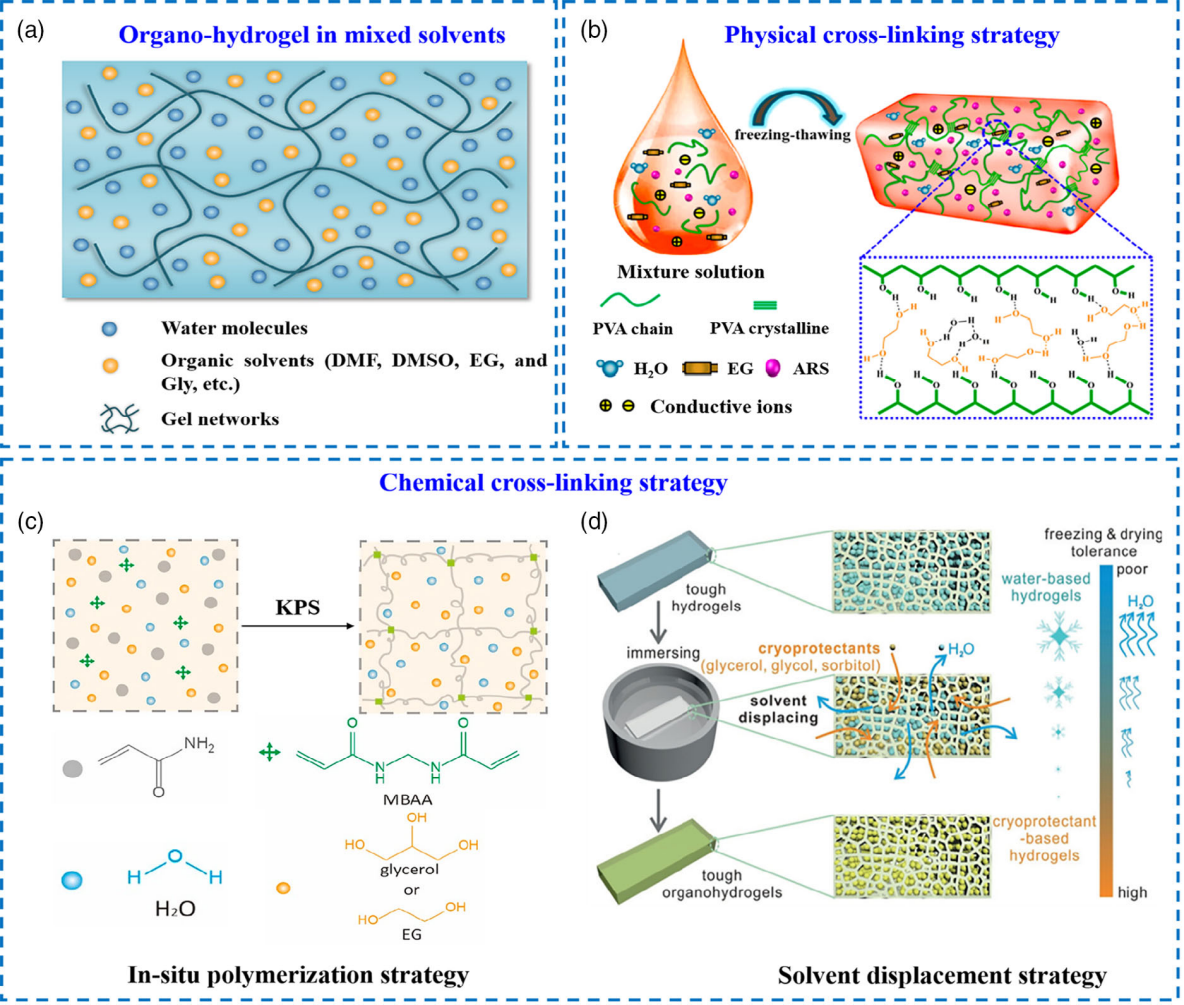
a) Organo‐hydrogel skeletons in hybrid (organic/water) dispersion medium. Reproduced with permission.^[^ ^37^ ^]^ Copyright 2021, Elsevier. b) Physical crosslinking strategy of organo‐hydrogels. Reproduced with permission.^[^ ^41^ ^]^ Copyright 2021, American Chemical Society. c) In situ polymerization strategy of organo‐hydrogels. Reproduced with permission.^[^ ^43^ ^]^ Copyright 2020, MDPI. d) Solvent displacement strategy of organo‐hydrogels. Reproduced with permission.^[^ ^46^ ^]^ Copyright 2018, Wiley‐VCH.

The term “organo‐hydrogel” was first raised by Guo et al. in 1997, which is composed of hydrophilic networks (poly(vinyl alcohol) (PVA) and cellulose) and mixed dispersion medium (water and dimethylformamide (DMF)).^[^ 35, 36 ^]^ Conceptually, organo‐hydrogel electrolytes represent that the hydrophilic and hydrophobic chain segments and salts exist in the same micro‐matrix containing the mixed solvents of organic solvents and water.^[^ ^37^ ^]^ Note that the utilized organic solvents should be highly soluble in water, such as DMF, ethylene glycol (EG), glycerol (Gly), sorbitol (Sor), dimethyl sulfoxide (DMSO), acetonitrile (ACN), and so forth (**Figure** **1**a). For the selection of an appropriate solvent, a high dielectric constant (ε > 20) is generally required to dissociate conductive ions and achieve stable solvation structures. To date, compared with purely homogeneous hydrogel electrolytes, considerable efforts have been devoted to designing and constructing organo‐hydrogel electrolytes due to their heterogeneous components and tailored structures.

### 2.1 Preparation Methods of Organo‐Hydrogel Electrolytes

The first reported organo‐hydrogel by right of an in situ gelation strategy was in 2017.^[^ ^38^ ^]^ This strategy introduces a binary solvent to the system before crosslinking the polymer network. In the energy storage application, diverse organo‐hydrogel electrolyte systems based on different dispersion media have been continuously exploited.^[^ ^39^ ^]^ Several crosslinking mechanisms, including physical and chemical crosslinking, can be used to prepare hydrophilic chain segments for gel materials.^[^ ^40^ ^]^

The physical crosslinking strategy refers to the gelation of synthetic or natural polymers in a solvent through physical entanglement and multiple non‐covalent interactions. Representatively, PVA‐based organo‐hydrogel electrolytes can be obtained by the freezing and thawing method to reconstruct hydrogen bonds.^[^ ^41^ ^]^ The presence of organic reagents increases the crosslinking sites, which can avoid repeated freezing–thawing cycles as in the preparation of hydrogels, thus significantly improving the gelation efficiency (Figure 1b). For instance, Rong et al. reported that EG molecules induced crystalline domains by forming massive hydrogen bonds with PVA chains in the PVA/LiCl/EG/H_2_O organo‐hydrogel electrolytes.^[^ ^26^ ^]^ In addition, gelatin‐based organo‐hydrogel electrolytes can also be prepared through physical crosslinking, where gelatin molecular chains appear in coil‐like shapes under initial heating (about 60 °C) and turn into triple helix structures on account of the hydrogen bonding interaction after cooling (4 °C).^[^ ^42^ ^]^ These gel networks are built based on green strategies that do not involve complex polymerization reactions.

In contrast, the chemical crosslinking strategy involves the utilization of free radical polymerization to introduce a dense covalent network into organo‐hydrogels, thereby ameliorating their mechanical robustness and environmental stabilities in electrolyte applications (Figure 1c).^[^ ^43^ ^]^ The factors to be considered in the polymerization process contain monomers, crosslinkers, initiators, and external stimulation conditions, such as light, heat, pH, atmosphere, etc.^[^ ^44^ ^]^ The typical polymer host skeletons obtained by polymerization reactions are polyacrylamide (PAM), poly(acrylic acid) (PAA), and sodium polyacrylate (PANa). Moreover, naturally derived polymers such as cellulose, sodium alginate, and chitosan are also employed in searching for environmentally friendly electrolytes owing to their abundant reserves and degradability. The hydrophilic functional groups of these polymers, such as hydroxyl groups, carboxyl groups, and amino groups, become crosslinking sites for covalent and ionic bonds. Although the polymer networks constructed with the aforementioned linkage structure deliver particular mechanical strength, they still cannot meet the requirements of commercial wearable devices in terms of toughness and elasticity. To overcome this issue, double networks are frequently adopted to produce organo‐hydrogels with ideal robustness through reversible energy dissipation.^[^ ^45^ ^]^

Generally, the organic/water mixed solvent systems with different polar components will significantly affect the polymerization process. Some gelation processes may be inhibited in the presence of specific organic agents. The cumbersome steps of partial polymerization reactions limit mass production. Hence, developing a versatile and mild strategy for preparing multitudinous organo‐hydrogels under different conditions is necessary. Motivated by this, a solvent replacement strategy was thus proposed, where a pre‐obtained hydrogel was immersed in a binary‐solvent system, and part of the water molecules was replaced by organic molecules based on the effect of osmotic pressure (Figure 1d).^[^ ^46^ ^]^ Benefiting from the synergistic effect of binary solvents, the functions of the organo‐hydrogels obtained by solvent replacement can be enriched by simply altering the soaking time, solvent concentration, and organic reagent types.^[^ 47, 48 ^]^ Significantly, the decrease in free water content leads to the contraction of hydrophilic chains and the formation of hydrophobic domains, transforming the brittle structures of original hydrogels into formidable networks.

Since the hydrogel precursors are easily dehydrated, organo‐hydrogel electrolytes can also be prepared by a two‐step method, where the pre‐fabricated hydrogels are freeze‐dried to aerogels immersed in mixed organic/water solutions containing conductive salts to reach swelling equilibrium. The intermediate aerogels can offer 3D porous network structures for sucking ample electrolytes and providing ion transport channels.^[^ ^49^ ^]^ Furthermore, the adjustment of cohesion in gels results in enhanced underwater adhesive performances, which is also expected to improve the interfacial interaction between electrolytes and electrodes. Based on these advantages, multifarious organo‐hydrogel electrolytes with superior environmental adaptability (frost resistance, water retention, adhesiveness, and mechanical stability) have been reported, dramatically increasing the varieties of such materials and broadening their applications.^[^ 50, 51, 52, 53 ^]^

### 2.2 Multiple Functions of Organo‐Hydrogel Electrolytes

In principle, the composition and structure of gel materials often determine their final performances and functions. Organo‐hydrogels possess both hydrophilic and lipophilic phases to accommodate highly complex environments, which is significantly superior to their hydrogel counterparts. This section mainly focuses on the desired functions of organo‐hydrogel electrolytes, such as temperature tolerance, mechanical stability, interfacial compatibility, and ionic conductivity.

#### 2.2.1 Temperature Tolerance

Free water molecules in homogeneous hydrophilic networks are susceptible to cold or high‐temperature environments, making hydrogels structurally susceptible to severe damage. To achieve the desired temperature tolerance, water crystallization and evaporation must be taken into account. Generally, water crystallization is the process where water molecules establish an infinite hydrogen bond network. Water evaporation is triggered by the lower vapor pressure in the surrounding environment. Hence, organic agents in organo‐hydrogels are expected to have stronger binding energy with water molecules and higher boiling point to partially replace free water and reduce the vapor pressure, thus enhancing frost/heat resistance.^[^ ^54^ ^]^ Thanks to predominant temperature tolerance, many fascinating organo‐hydrogel electrolytes have been continuously prepared to achieve stable and long‐lasting energy storage.^[^ ^55^ ^]^

**Figure 2:**
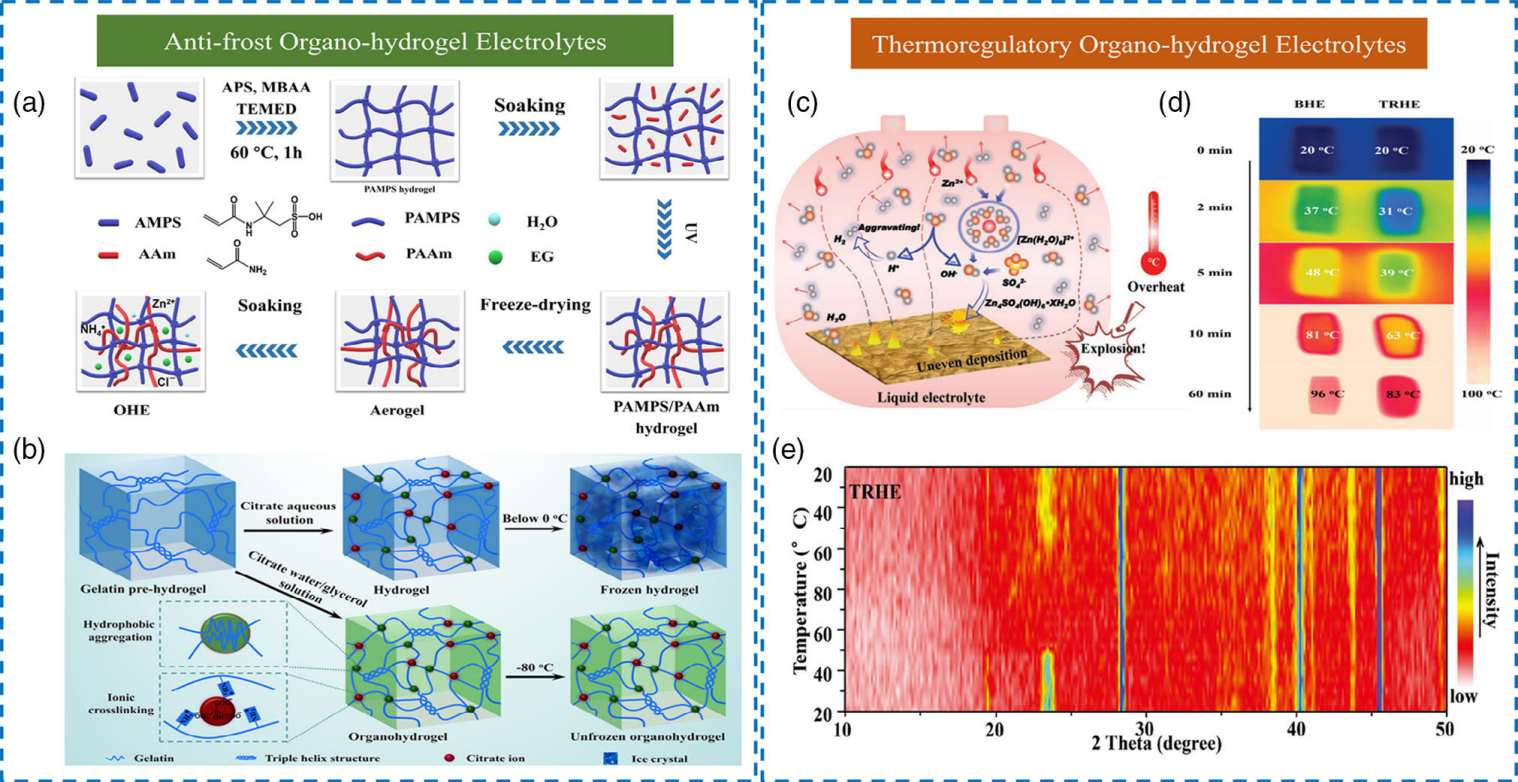
a) Synthesis route of the ethylene glycol (EG)‐modified organo‐hydrogel electrolyte. Reproduced with permission.^[^ ^57^ ^]^ Copyright 2022, Elsevier. b) The formation of the gelatin supramolecular organo‐hydrogels with anti‐freezing properties. Reproduced with permission.^[^ ^50^ ^]^ Copyright 2019, American Chemical Society. c) Thermal stable problems in traditional liquid electrolytes. d) Infrared thermal images. e) In situ X‐ray diffraction (XRD) characterizations of the organo‐hydrogel electrolyte during heating and cooling. c–e) Reproduced with permission.^[^ ^33^ ^]^ Copyright 2022, Wiley‐VCH.

Nowadays, EG is the cheapest and most commonly used organic protective solvent, which can act as a strong “water blocker” to allow the freezing point of the electrolyte below −40 °C to avoid water crystallization. In this respect, a cellulose/NaCl organo‐hydrogel electrolyte was reported by employing the EG/H_2_O binary solvent, which showed the lowest freezing point down to –54.3 °C at the EG volume percentage of 60%.^[^ ^56^ ^]^ In another case, the introduction of EG significantly minimized the content of accessible solvents by the solvation effects (hydrogen bonds and coordination bonds) among solvent molecules, conductive salts, and polymer backbone (**Figure** **2**a). The weight loss of this organo‐hydrogel at 80 °C was only 19%, obviously lower than that of the corresponding hydrogel (76%), suggesting the improved anti‐drying and water‐retention performance.^[^ ^57^ ^]^ These results collectively demonstrated that the adjustment of EG content contributed to the regular operation of the quasi‐solid‐state electrolytes under all‐climate conditions. Compared with hygroscopic EG, Gly possesses a higher boiling point, lower vapor pressure, and better freezing resistance. A Gly/water binary solvent has been applied for fabricating a gelatin‐based supramolecular organo‐hydrogel, which could be twisted without fracture at −80 °C and retained highly transparent even in liquid nitrogen (Figure 2b).^[^ ^50^ ^]^ Meanwhile, Gly hampered the evaporation of water molecules via forming firm hydrogen bonds, guaranteeing long‐term stability in real‐world applications. As expected, the weight of this organo‐hydrogel displayed a subtle variation within 7 days and almost 90 wt% of the original weight retention could be accomplished.

To further reduce the freezing point of the hybrid solution system, DMSO is another promising organic solvent owing to its excellent chemical stability and high polarity for utilization in anti‐freezing electrolytes. For instance, a novel PVA/graphene/H_2_SO_4_/DMSO/H_2_O organo‐hydrogel electrolyte was constructed to support the normal operation of a flexible supercapacitor at −65 °C.^[^ ^32^ ^]^ When the molar ratio of DMSO to water exceeded 3:2, the freezing point of the mixed solvent reached −65 °C. The ionic conductivity of the fabricated quasi‐solid‐state electrolyte at this low subzero temperature remained as high as 1.0 S m^−1^ because of the prominent decrease of the freezing point in the DMSO/H_2_O mixture. To elucidate the intermolecular interaction of the DMSO–H_2_O system, density functional theory (DFT) analysis demonstrated that the hydrogen bonding interactions between DMSO and H_2_O molecules (−14.089 kcal mol^−1^) were more substantial than that between H_2_O molecules (−7.172 kcal mol^−1^). Afterward, the obtained binding energies of DMSO/H_2_O to polymer backbones (−61.040 kcal mol^−1^) were also higher than that of DMSO/H_2_O.^[^ ^58^ ^]^ Hence, DMSO can serve as a strong hydrogen bonding acceptor to destroy the pristine water configuration and further affect the interactions among polymer segments. The reinforced network by tightly bounding H_2_O molecules around the C–S groups would form ordered ion migration channels.

Although water loss is greatly mitigated in organo‐hydrogel electrolytes, dehydration inevitably occurs under arid environments or after long‐term cycles. Ideally, a self‐regenerating organo‐hydrogel is urgently desired to be developed so that it can regenerate to its original state. In such a system, owing to the low vapor pressure, the partially dehydrated organo‐hydrogel can spontaneously absorb water molecules from the surrounding environment and return to its original state. Since the ability of organo‐hydrogels to absorb water was discovered in the previously reported study,^[^ ^59^ ^]^ the recovery of water content and the maintenance of stability can be achieved by adjusting the proportion of organic agents and the humidity of the ambient environment. Furthermore, the most extreme case of water evaporation is thermal shock, which means that all the accumulated heat in batteries cannot be released within a specified time, resulting in an instantaneous rise in temperature and rapid water runaway (Figure 2c). Thermal shock is thus a critical topic in the design of organo‐hydrogel electrolytes, closely related to the operational stability and safety of devices. To mitigate the thermal shock while maintaining the electrochemical performance, Meng et al. devised a thermoregulatory organo‐hydrogel electrolyte based on agarose backbones (AGr) and poly(ethylene glycol) (PEG) chains, showing excellent thermal energy storage from the monitoring of infrared thermal imagers (Figure 2d).^[^ ^33^ ^]^ When the heating temperature increased from 20 to 50 °C, the PEG phase reversed from crystalline to amorphous, which was confirmed by the in situ X‐ray diffraction (XRD) characterization (Figure 2e). In addition, the infrared thermal images also showed that adding PEG into solvent slowed down the heating rate of the organo‐hydrogel electrolyte, indicating its heat‐storage capability while the temperature was raised from room temperature to 100 °C. Therefore, this phase transition mechanism with endothermic effects could act as a thermal buffer to avoid sudden overheating and explosion.

#### 2.2.2 Mechanical Stability

Flexible energy‐supplied electronic devices are often anticipated to deliver extraordinary mechanical stability, such as high strength, excellent stretchability, and good self‐healing behavior. These properties are mainly related to the crosslinking density among all components in the gel networks. Considering the loose crosslinking of traditional hydrogels attributed to high pure water content, the organic agents can interact with water and polymers via a large number of hydrogen bonds in organo‐hydrogels, which can adjust the bridging and crosslinking degree of polymer networks, thereby enhancing the mechanical properties in response to complicated external stimuli.^[^ ^60^ ^]^ Generally, the synergistic effect of the hydrophilic and lipophilic components in the sophisticated organo‐hydrogels can achieve precise mechanic regulation. The organic solvents in organo‐hydrogels should form plenty of hydrogen bonds with polymers in the network, which can increase the crosslinking density and promote the bridging of polymer chains, thus enhancing the mechanical properties. Crystalline domains of polymers can be induced to form as high‐functionality crosslinkers to toughen the organo‐hydrogels via the crack pinning effect. Additionally, the design of double networks is also an effective strategy to improve mechanical performance.

**Figure 3:**
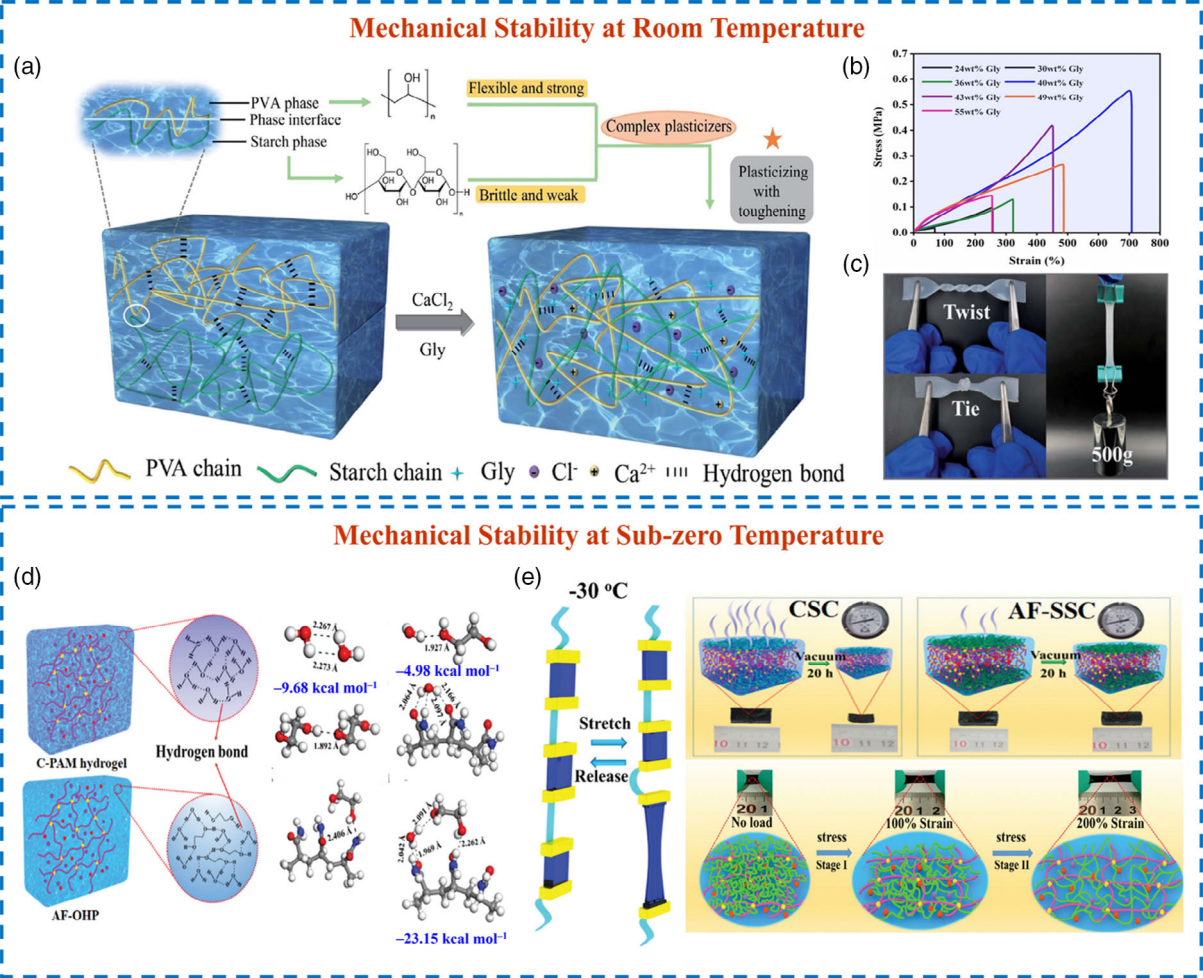
a) Synthetic mechanism of the (poly(vinyl alcohol) (PVA)/starch/CaCl_2_/Gly/H_2_O organo‐hydrogel electrolyte. b) Tensile *σ*–*ε* curves of organo‐hydrogels using different Gly contents. c) Deformation tests. a–c) Reproduced with permission.^[^ ^62^ ^]^ Copyright 2021, Royal Society of Chemistry. d) Hydrogen bonding between different molecules. e) Schematic illustration of the organo‐hydrogel under –30 °C, vacuum environment, and stretching treatment. d,e) Reproduced with permission.^[^ ^63^ ^]^ Copyright 2021, Royal Society of Chemistry.

In organo‐hydrogel electrolyte energy storage devices, gel materials can be used as electrolytes and separators, which can address the leakage issue of aqueous electrolytes under external tensile strain.^[^ ^61^ ^]^ Based on the intrinsic mechanical merits of organo‐hydrogel electrolytes, stretchable supercapacitors or batteries can be assembled through the shape transformations of organo‐hydrogels. For example, Lu et al. prepared a starch/PVA dual dynamic supramolecular polymer network, which was effectively strengthened by adding Gly and CaCl_2_ into the system (**Figure** **3**a).^[^ ^62^ ^]^ Since starch and PVA both exhibited semi‐crystalline nature and were partially compatible, the surface of the initial starch/PVA hydrogel was rough and uneven. Gly with three hydroxyl groups could provide enough crosslinking sites by constructing hydrogen bonds with starch and PVA chains, thus promoting the compatibility of heterogeneous polymers. The highest tensile strength and elongation at break were 0.55 ± 0.06 MPa and 704% ± 12%, respectively, when the content of Gly in the organo‐hydrogel electrolyte was adjusted to 40 wt% (Figure 3b). Moreover, the mechanical properties including deformation flexibility, toughness, Young's modulus, elasticity, and recovery performances were all significantly enhanced due to the existence of favorable dynamic hydrogen bonds (Figure 3c). Nowadays, as the freezing point of the gel electrolytes gradually dropped below the ice point, stretchability and flexibility at low temperatures must be considered to ensure the normal operation of solid‐state devices in extreme circumstances. Jin et al. immersed the PAM hydrogel into an EG/water/H_2_SO_4_ mixed solution to prepare an anti‐freezing organo‐hydrogel electrolyte.^[^ ^63^ ^]^ As illustrated in Figure 3d, EG bound water molecules tightly together into stable clusters, and the EG–water mixture exhibited stronger interactions with PAM chains (–23.15 kcal mol^−1^) than sole water (–9.68 kcal mol^−1^) or EG (–4.98 kcal mol^−1^). In this system, water molecules were used as a link point with the hydroxyl groups of EG and the carbonyl groups of PAM networks to endow high structural integrity (Figure 3e), warranting the suppressed water crystal and good mechanical performances at –30 °C. Specifically, the supercapacitor fabricated by this organo‐hydrogel electrolyte showed reversible stretchability at –30 °C, where its stress–strain (*σ*–*ε*) curves were close to the measured results at room temperature. Meanwhile, the electrochemical measurement results including cyclic voltammetry (CV) curves and galvanostatic charge/discharge (GCD) profiles showed no significant change even after 100 cycles of repeated stretching in a low‐temperature environment.

**Figure 4:**
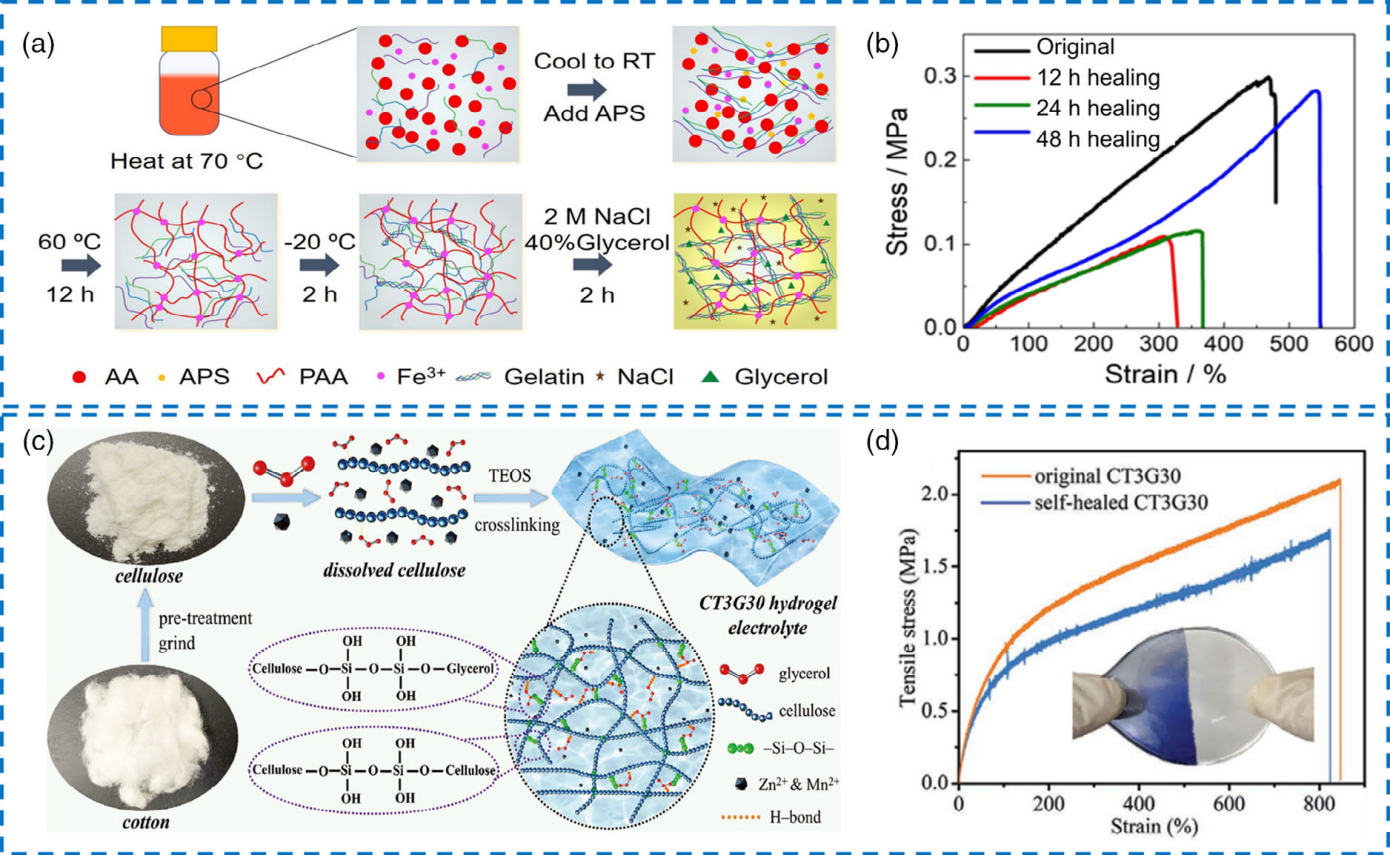
a) Schematic diagram of the preparation methods for the gelatin/PAA/NaCl/Gly/H_2_O organo‐hydrogel electrolytes. b) Tensile *σ*–*ε* curves of the organo‐hydrogel under different self‐healing times. a,b) Reproduced with permission.^[^ ^42^ ^]^ Copyright 2020, American Chemical Society. c) Synthesis schematic of the cellulose/TEOS‐based organo‐hydrogel electrolyte. d) Tensile *σ*–*ε* curves of the original and self‐healed organo‐hydrogels. c,d) Reproduced with permission.^[^ ^75^ ^]^ Copyright 2022, Wiley‐VCH.

Self‐healing capacity is an important parameter to assess the ability of organo‐hydrogel electrolytes to repair some unexpected damages. The self‐healing mechanism is achieved through the diffusion and redistribution of molecules and intrinsically reversible chemical bonds, which can be divided into non‐covalent crosslinking and dynamic covalent crosslinking.^[^ 64, 65 ^]^ First, non‐covalent crosslinking methods primarily include hydrogen bonding, ionic and metal–ligand coordination, and host–guest interaction. For example, Fang et al. prepared a versatile tough organo‐hydrogel electrolyte by soaking a gelatin/Fe^3+^‐crosslinked PAA dual dynamic supramolecular network into a NaCl/Gly/water solution (**Figure** **4**a).^[^ ^42^ ^]^ After being cut into halves, the organo‐hydrogel electrolyte was rejoined as a whole and subsequently stretched to approximately 250% strain on account of the synergic effect of hydrogen bonds induced by Gly and electrostatic interactions between gelatin (–NH_3_^+^) and PAA (–COO^−^). Tensile curves obviously demonstrated that the recovery of mechanical properties became more desirable with the extending self‐healing time, and the organo‐hydrogel samples recovered almost completely after entirely 48 h of exposure at room temperature (Figure 4b). Such mechanical self‐healing performance met the requirement of assembling self‐healing stretchable micro‐supercapacitors. As for the covalent crosslinking methods, different reversible covalent systems such as Schiff base bond recombination,^[^ ^66^ ^]^ Diels–Alder reaction,^[^ 67, 68, 69, 70 ^]^ acyl hydrazone re‐bonding,^[^ ^71^ ^]^ alkoxyamine,^[^ ^72^ ^]^ borate ester bonds,^[^ 73, 74 ^]^ siloxane bonds,^[^ ^75^ ^]^ and disulfide bonds^[^ 76, 77 ^]^ have been investigated for realizing self‐healing capabilities. In this regard, the cellulose obtained from cotton was crosslinked by tetraethyl orthosilicate (TEO) to prepare a novel organo‐hydrogel electrolyte containing ZnSO_4_, MnSO_4_, and Gly. TEO was hydrolyzed to form Si(OH)_4_, binding to hydroxyl groups in cellulose and Gly through siloxane bonds, generating a 3D porous structure (Figure 4c).^[^ ^75^ ^]^ The self‐healing efficiency of this organo‐hydrogel electrolyte reached 82.6%, which was mainly ascribed to a large number of reversible hydrogen bonds and Si—O—Si bonds with good mobility inside the gel (Figure 4d).

#### 2.2.3 Interfacial Compatibility

The firm contact of an organo‐hydrogel electrolyte with two electrodes can make it possess the potential to be applied in flexible energy storage devices, which means that organo‐hydrogels should display good adhesion.^[^ ^78^ ^]^ Comparatively, the adhesion of hydrogels to various substrates, such as scotch tape, requires external assistance.^[^ 79, 80 ^]^ This problem increases the complexity of the packaging process and is not conducive to ions/electrons transfer at the interface between electrodes and electrolytes. Therefore, there is a crucial requirement for developing organo‐hydrogel electrolytes with superior contact properties, which requires that the organo‐hydrogels have good interfacial compatibility with electrodes without extra assistance to achieve integrated high‐performance devices. The preferable interfacial stability can be achieved by the introduction of adhesive components or specific functional groups in the polymer. These functionalized organo‐hydrogel electrolytes can further form covalent or non‐covalent interactions with electrode surfaces, providing channels for ion transfer.

**Figure 5:**
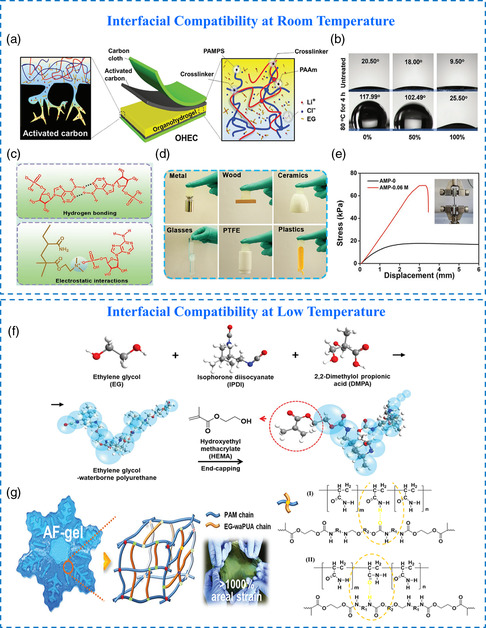
a) Diagram of ion diffusion from electrolytes to electrodes. b) Contact angles of water on the organo‐hydrogels. a,b) Reproduced with permission.^[^ ^29^ ^]^ Copyright 2020, Wiley‐VCH. c) Diagram of the interaction inside the organo‐hydrogel electrolyte. d) Photographs of the organo‐hydrogels adhering to different substrates. e) Lap shear test of the solid‐state supercapacitor. c–e) Reproduced with permission.^[^ ^34^ ^]^ Copyright 2021, Wiley‐VCH. f) Scheme of the EG‐waPUA gel. g) Hypothetical molecular model of this anti‐freezing gel, which could be stretched over 1000% in area. f,g) Reproduced with permission.^[^ ^81^ ^]^ Copyright 2019, Royal Society of Chemistry.

It has been reported that the surface of commercial activated carbon electrodes delivers hydrophobicity with a contact angle of 119.99°. With the gradual increase of EG content in the poly(2‐acrylamido‐2‐methylpropanesulfonic acid)/polyacrylamide (PAMPS/PAM) dual‐network system (**Figure** **5**a), the surface properties of the organo‐hydrogel electrolyte would change fundamentally at 80 °C, where the surface contact angle increased from 20.5° to 117.99° indicating the transformation of hydrophilicity to hydrophobicity (Figure 5b).^[^ ^29^ ^]^ In this case, ions were quickly transferred from the hydrophobic interface of the electrolyte to the carbon‐based electrode layer, which significantly reduced interfacial transfer resistance, optimized current distribution, and improved flow performance. In addition, Zhang et al. reported a nucleotide tackifier adhesive organo‐hydrogel electrolyte, where negatively charged adenosine monophosphate (AMP) constructed electrostatic interactions with positively charged poly(acrylamide‐*co*‐2‐(dimethylamino)ethylmethacrylate)/gelatin (P(AM‐*co*‐DMAEMA)/gelatin) chains.^[^ ^34^ ^]^ The large functional groups (–N=, –NH_2_, and nitrogen heterocycle) in AMP endowed the enhanced adhesion of the organo‐hydrogel to diverse materials (Figure 5c). Further, superior adhesion was essential for organo‐hydrogel electrolytes to hold close contact with electrodes and avoid delamination/displacement between multilayered components under external stress (Figure 5d). The optical microscopy presented a sandwich‐structured supercapacitor based on this AMP‐tackified organo‐hydrogel electrolyte with no apparent interfacial boundary. The lap shear test was further employed to validate that the shear adhesion strength of the supercapacitor reached 69.2 kPa (Figure 5e). Such robust interfacial adhesion also reduced interfacial contact resistance and optimized capacitive performance.

Furthermore, the interfacial stability at low temperatures is also pivotal to ameliorating the environmental adaptability of batteries. It should be noted that hydrogels will become hard and brittle after freezing, which makes the interfacial adhesion between electrolytes and electrodes decrease and the interfacial charge transfer resistance increase. In view of this problem, Mo et al. combined EG‐based waterborne anionic polyurethane acrylates (EG‐waPUA) and PAM to design a novel dual crosslinked organo‐hydrogel electrolyte (Figure 5f).^[^ ^81^ ^]^ On the one hand, EG‐waPUA and PAM worked together to lock water molecules and disrupted the generation of crystal lattices, ensuring superior anti‐freezing properties and high adhesion of the organo‐hydrogel electrolyte. The interaction energy of both EG‐waPUA and PAM with water molecules was thrice that of water and EG according to the results of DFT calculations. On the other hand, the dual crosslinked polymer chains acted as stress buffers to dissipate energy and homogenize the gel network (Figure 5g). Consequently, the assembled Zn–MnO_2_ battery still showed good flexibility without transforming into an ice‐like solid at –20 °C, and no interfacial voids were found between the electrode and electrolyte under external force. Benefiting from the stable interfacial chemistry, there was no significant change in the GCD curves at different bending angles, while all the voltage platforms were also maintained between 1.35 and 1.6 V. In the application of Zn–air batteries, a stable electrode–electrolyte interface is also able to ensure efficient mass transport and rapid ion/electron transfer. Pei et al. found that the increased polarity of the terminal functional groups in polymer chains enhanced the interaction with water molecules.^[^ ^82^ ^]^ The rationally designed organo‐hydrogel electrolyte delivered good contact with a stereoscopic air cathode to form high‐performance triple‐phase interfaces, significantly enriching reactive interfaces and facilitating oxygen transport.

#### 2.2.4 Ionic Conductivity

In actual application, organo‐hydrogel electrolytes with high ionic conductivity are crucial for developing flexible electronics. In the experiment, the ionic conductivity (*σ*, S cm^−1^) can be obtained by sandwiching organo‐hydrogel with two symmetric metal (stainless steel, titanium, etc.) foils and measuring it through electrochemical impedance spectroscopy (EIS). The calculation formula is as follows: *σ* = *L*/*RA*, where *L* (cm), *R* (Ω), and *A* (cm^2^) represent the electrolyte thickness, electrode cross‐section area, and bulk ohmic resistance.^[^ ^83^ ^]^ Besides, the Arrhenius formula (*σ* = *σ*_0_ exp(−*E*_a_/*kT*)) can also be utilized to study the ion transport mechanism of polymer gel electrolytes and reveal the relationship between ion diffusion and temperature.^[^ ^84^ ^]^

**Figure 6:**
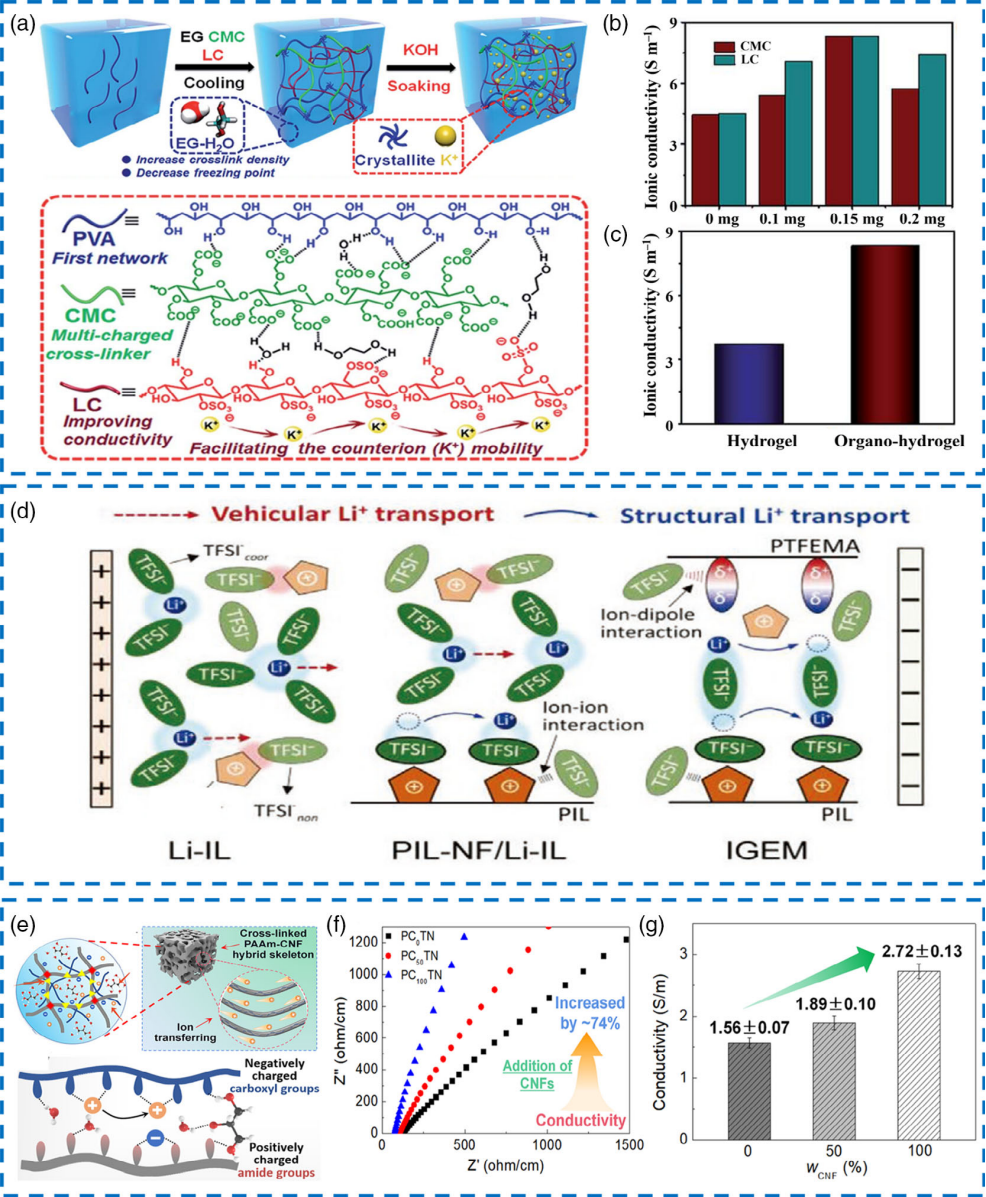
a) Illustration of the PVA/carboxymethyl cellulose sodium (CMC)/*λ*–carrageenan gels (LC) organo‐hydrogel electrolyte network. b) Ionic conductivity of the organo‐hydrogels with different CMC contents. c) Comparison of ionic conductivity between the hydrogel and organo‐hydrogel. a–c) Reproduced with permission.^[^ ^85^ ^]^ Copyright 2022, Royal Society of Chemistry. d) Mechanism of ionic diffusion. Reproduced with permission.^[^ ^86^ ^]^ Copyright 2021, Wiley‐VCH. e) Illustration of the interaction between the gel skeleton and electrolyte ions. f) Nyquist plots of the organo‐hydrogels with various CNF contents. g) Ionic conductivities of different organo‐hydrogels. e–g) Reproduced with permission.^[^ ^87^ ^]^ Copyright 2021, American Chemical Society.

In organo‐hydrogel electrolytes, ion concentration, organic reagent content and motion characteristics of polymer chain segments jointly determine the ionic conductivity. Unfortunately, there is a discordant contradiction between mechanical properties and ionic conductivity in polymer networks, which suggests that an increase in the crosslinking degree may cause a decrease in the ion migration rate. Accordingly, Yang et al. incorporated *λ*‐carrageenan gels (LC) with negatively charged sulfate groups, carboxymethyl cellulose sodium (CMC) with carboxylate anions, and EG into the origin PVA matrix forming a heterogeneous branched network structure (PVA–CMC–LC) and giving rise to a fixed K^+^ channel (**Figure** **6**a).^[^ ^85^ ^]^ After optimizing the content of CMC and LC, the highest ionic conductivity of PVA–CMC–LC at room temperature reached 8.3 S m^−1^ (Figure 6b), while the crosslinking degree was also enhanced due to the hydrogen bonding interaction among EG, PVA, and LC. The as‐obtained PVA–CMC–LC organo‐hydrogel electrolyte exhibited higher ionic conductivity than the corresponding hydrogel electrolyte (Figure 6c), which was reasonably credited to more water molecules locked in the organo‐hydrogel. EG penetrated the gel network in the soaking process, increasing the inhalation amount of KOH and water, thus resulting in high water retention. Further, the anti‐freezing ability and thermal stability were significantly improved, and this organo‐hydrogel electrolyte delivered excellent ionic conductivity of 3.18 and 9.67 S m^−1^ at −40 °C and 60 °C, respectively.

Charge transfer in organo‐hydrogel electrolytes is based on carrier migration under an external electric field, which is currently dominated by the vehicular diffusion mechanism. Vehicular transport is associated with the first solvent shell of ions, where ions often fail to migrate in a particular direction and are also subjected to the interference of polymer chains (Figure 6d).^[^ ^86^ ^]^ In contrast, structural diffusion, also known as Grotthuss diffusion, means that unrelated ions hop from one coordination site to another, which will facilitate rapid ion migration.^[^ ^87^ ^]^ In the practical situation, organo‐hydrogel electrolytes with high ionic conductivity can be achieved by selecting appropriate ions and terminal functional groups. For instance, Wei et al. incorporated cellulose nanofibrils (CNF), tannic acid (TA), NaCl, and Gly‐water solvent into the covalently crosslinked PAM network to induce supramolecular interactions.^[^ ^88^ ^]^ CNFs with negatively charged surfaces were obtained through 2,2,6,6‐tetramethylpiperidine‐1‐oxyl (TEMPO)‐treated and high‐pressure homogenization procedure. CNFs could serve as rigid skeletons, enhancing their mechanical properties and offering abundant counterion hopping sites for quick ion transport (Figure 6e). With the increased CNF content, the micron‐level uniform channels appeared inside the gel, full of Na^+^ and Cl^−^ ions dispersed in Gly‐water mixed solvent (Figure 6f). As predicted above, after further adjustment of NaCl content, the ionic conductivity was increased to 2.72 S cm^−1^ (Figure 6g). Otherwise, it would be inclined to form ion clusters or pairs, resulting in the saturation of ion migration. Conductivity essentially means the movement of free ions. The ionic conductivity of organo‐hydrogel electrolytes can be affected by the adjustment of conductive salt concentration, the grafting of charged functional groups in the polymers, and the participation of organic solvents in the solvated structure.

## 3 Organo‐Hydrogel Electrolytes for Supercapacitors

Supercapacitors store energy mainly through either surface electrostatic adsorption of electrolyte ions, classified as electrical double‐layer capacitors, or rapid surface redox reactions, which belong to pseudo‐capacitors.^[^ 89, 90, 91 ^]^ Although supercapacitors possess the advantages of fast charge/discharge rates, long cycling life, and environmental benignity, the low energy density restricts their further development in practical applications. Electrolytes determine the two critical parameters of capacitance and operating voltage which closely correlates with the energy density of supercapacitors.^[^ ^92^ ^]^ To achieve all‐around superior performance, organo‐hydrogel electrolytes have been harvesting continuous research attention owing to their versatilities, high chemical stability, decent ionic conductivity, and remarkable mechanical properties. Furthermore, supercapacitors with appealing additional functions (temperature tolerance, self‐healing ability, stretchability, etc.) that arise from organo‐hydrogels could also be assembled in complicated environments. In the following section, the latest advances in flexible supercapacitors utilizing organo‐hydrogel electrolytes are discussed in depth.

### 3.1 Flexible Organo‐Hydrogel Electrolytes for Supercapacitors

**Figure 7:**
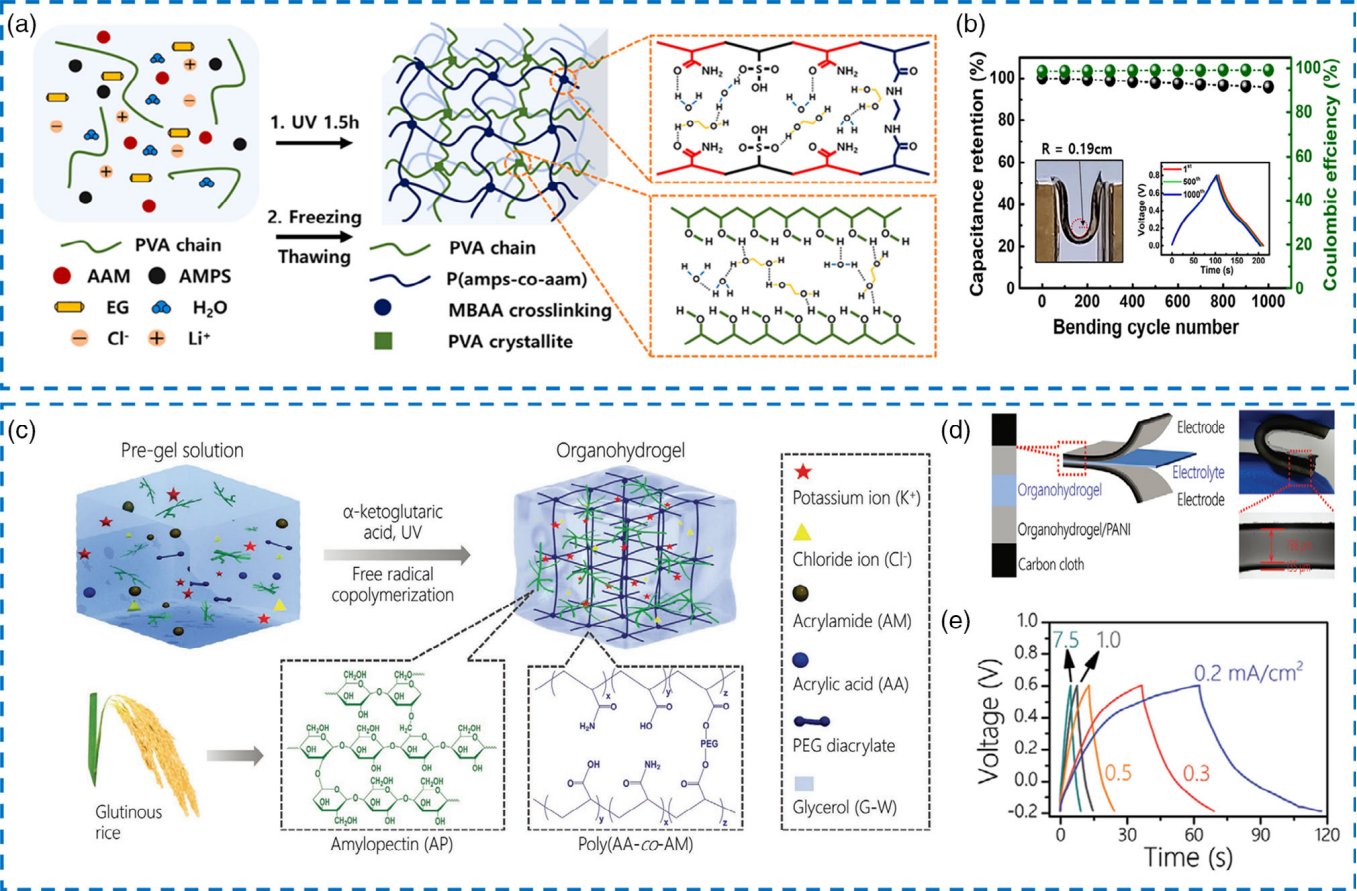
a) Schematic illustration of the PAMPs‐*co*‐PAAM/PVA‐based organo‐hydrogels. b) Capacitance retention under 1000 bending repeated cycles with a bending radius of 0.19 cm at 1 A g^−1^. a,b) Reproduced with permission.^[^ ^97^ ^]^ Copyright 2022, Elsevier. c) Scheme of the preparation for glutinous‐rice‐inspired organo‐hydrogels. d) Illustration of the flexible all‐in‐one supercapacitor containing a sandwiched organo‐hydrogel electrolyte. e) Galvanostatic charge/discharge (GCD) profiles under different current densities. c–e) Reproduced with permission.^[^ ^28^ ^]^ Copyright 2022, Wiley‐VCH.

As the most commonly used polymer skeleton material, PVA has been applied in organo‐hydrogels for supercapacitor electrolyte materials due to their nontoxicity, low price, and stable chemical structure. However, some inherent shortcomings in PVA indeed give rise to practical problems.^[^ ^93^ ^]^ For example, the long carbon chains containing insufficient hydrophilic groups in PVA may weaken binding energy to water molecules. Under the circumstances, water molecules in gel electrolytes were prone to volatilization and the cycle stability would deteriorate during the long‐term running process. After solidification, the mechanical stiffness and unsatisfactory self‐discharge problem of PVA‐based electrolytes also limited their application in supercapacitors.^[^ 94, 95, 96 ^]^ Consequently, several endeavors were underway to design advanced organo‐hydrogel electrolytes with superior mechanical flexibility for high‐performance supercapacitors. Jung et al. employed a UV‐induced radical polymerization strategy to prepare a poly(2‐acrylamido‐2‐methyl‐1‐propanesulfonic acid–acrylamide)/PVA (PAMPS‐*co*‐PAM/PVA) polymer network containing LiCl conductive salt and EG/water binary solvent, which was further used as an organo‐hydrogel electrolyte in a flexible supercapacitor (**Figure** **7**a).^[^ ^97^ ^]^ The organo‐hydrogel using the EG/water mixed solvent displayed a higher tensile strength and superior stretchability than the hydrogel with pure water solvent. Specifically, the prepared organo‐hydrogel was stretched up to 648%, while the hydrogel was only 379%. Correspondingly, the tensile strength of the organo‐hydrogel (75.5 kPa) was also higher than that of the hydrogel (49.6 kPa). The loading–unloading measurement further demonstrated the highly stable elasticity of this organo‐hydrogel. These excellent mechanical properties could probably be ascribed to the presence of hydrogen bonds between EG and polymers. The quasi‐solid‐state supercapacitor assembled with carbon nanotube/polyaniline (CNT/PANI) electrode and the above‐mentioned organo‐hydrogel electrolyte delivered a maximum specific capacitance up to 147.0 F g^−1^ at 0.25 A g^−1^. Besides, the electrochemical performance was almost not degraded after the bending radius of the flexible supercapacitor decreased to 0.19 cm. After 1000 repeated bending deformations at a bending radius of 0.19 cm, capacitance retention and Coulombic efficiency both reached 98%. The supercapacitor sustained nearly 85% of the original capacitance after 5000 consecutive charging/discharging cycles at different bending radii from 0.82 to 0.19 cm, which fully guaranteed cyclic stability (Figure 7b). In other aspects, a triple‐network structure was formed by incorporating sodium alginate (SA) and cellulose nanofibrils (CNFs) into PVA, which would significantly increase the fracture strain and tensile strength up to 611.5% and 212.7 kPa, respectively.^[^ ^98^ ^]^ The resulting supercapacitor also exhibited superior electrochemical stability under different bending deformations. Additionally, the organo‐hydrogel electrolyte prepared by incorporating PVA, alizarin red S (ARS), and H_2_SO_4_ into EG/H_2_O binary solvent could also endure various deformations such as stretching, twisting, and knotted stretching. It was able to lift 500 × g weights even at −37 °C.^[^ ^45^ ^]^ Similarly, the assembled supercapacitor using active organo‐hydrogel electrolyte still maintained high capacitance after long‐term bending‐releasing cycles.

Apart from physically crosslinked PVA, other organo‐hydrogels have been reported as alternative electrolyte materials. Mechanical robustness and flexibility are essential for organo‐hydrogel electrolytes to be used in supercapacitors.^[^ ^99^ ^]^ For example, a semi‐interpenetrating polymer electrolyte was prepared by Zhou et al. by incorporating amylopectin (AP) into a chemically crosslinked copolymer network of PAA and PAM in the Gly/water binary solvent containing KCl (Figure 7c).^[^ ^28^ ^]^ Among all components, AP significantly affected the mechanical properties of the resultant organo‐hydrogel. When AP content was below 1.87 wt%, the increase of AP led to the enhancement of stretchability and decline of strength. Above the AP content of 1.87 wt%, the further increase of AP would induce a decrease in stretchability. The most considerable elongation at a break of 1089% was recorded. 20 cycles of nearly overlapping hysteresis loops evidenced the excellent energy dissipation and fatigue resistance of such organo‐hydrogel under loading–unloading tests. Since there were abundant hydroxyl groups in AP, the adhesiveness of this organo‐hydrogel to the substrate could also be enhanced through supramolecular interactions. After adjusting AP content to 3.73 wt%, the adhesion strength was increased to 21.16 kPa. It should also be noted that homogeneously dispersed Gly weakened the hydration effect of polymers and expose functional groups, thus promoting the enhancement of the gel network. As shown in Figure 7d, one all‐in‐one supercapacitor coupled with two polyanilines (PANI) electrodes and one poly(AA‐*co*‐AM)/AP/KCl/Gly/water organo‐hydrogel electrolyte was easily bent to 180°, where the thickness of electrode and electrolyte was about 135 and 788 μm, respectively. GCD profiles from 0.2 to 7.5 mA cm^−2^ all displayed triangle shapes while the specific areal capacitance was 14.30 mF cm^−2^ at 0.2 mA cm^−2^ (Figure 7e). Water electrolysis reduces ESPW, not only limiting the energy density of the storage device, but also leading to cyclic stability deterioration due to the gradual accumulation of gas. This problem can be avoided using a “water‐in‐salt” strategy, where a high concentration of salt can assist in inhibiting the decomposition of water. Polyurethane (PU) with abundant hydrophilic/lipophilic segments is well known for excellent biocompatibility, superb mechanical properties, and self‐adhesiveness, and is expected as an ideal elastic matrix. While introducing a high concentration of H_2_O/ACN‐NaClO_4_ into amphiphilic PU frameworks, the prepared electrolyte delivered a wide voltage window (≈2.3 V) and good resilience. Plastic deformation is stable at ≈4.8%, and the minimum energy loss coefficient after 20 cycles is only 0.06.^[^ ^100^ ^]^

A combination of multifarious polymers is also an effective strategy to improve the mechanical properties of organo‐hydrogels. In this regard, Huang et al. synthesized PVA/PAMAA polymeric networks containing CaCl_2_ salt via free radical polymerization reaction with the initiation of UV by dissolving AA, AM, and PVA into a DMSO–H_2_O mixed solvent.^[^ ^101^ ^]^ The hydroxyl and amide groups in this system were copolymerized to realize the reconstruction of macromolecular chains and further endowed the organo‐hydrogel with superior viscoelastic flexibility. The as‐prepared organo‐hydrogel possessed the highest strain of 1500% at a strength of 270 kPa. Based on its desirable electrochemical properties and mechanical toughness, the flexible supercapacitor constructed by this organo‐hydrogel electrolyte withstood various deformation treatments without noticeable damage. Under the external stress of stretching, bending, and compressing, all the manufactured supercapacitors maintained almost 100% of the initial capacitance, and even reached over 120% of the initial value at compressing stress of 50 kPa. Unlike traditional supercapacitors, for the construction of devices with superior mechanical flexibility, the significance of quasi‐solid‐state organo‐hydrogel electrolytes was accomplished and highlighted, as they were both indispensable electrochemical components and efficient mechanical buffers.

### 3.2 Anti‐Freezing Organo‐Hydrogel Electrolytes for Supercapacitors

Traditional hydrogel electrolytes with water as the medium tend to freeze below the freezing point, which will seriously degrade the original properties (ionic conductivity, capacitance, and elasticity) of supercapacitors.^[^ ^102^ ^]^ Thus, devices are restrictively applied in many low‐temperature scenarios (high altitude or high latitude areas). To satisfy the application of supercapacitors at very low temperatures, electrolytes can be improved by breaking hydrogen bonds among free water molecules or strengthening the interaction between polymer chains and water molecules.

**Figure 8:**
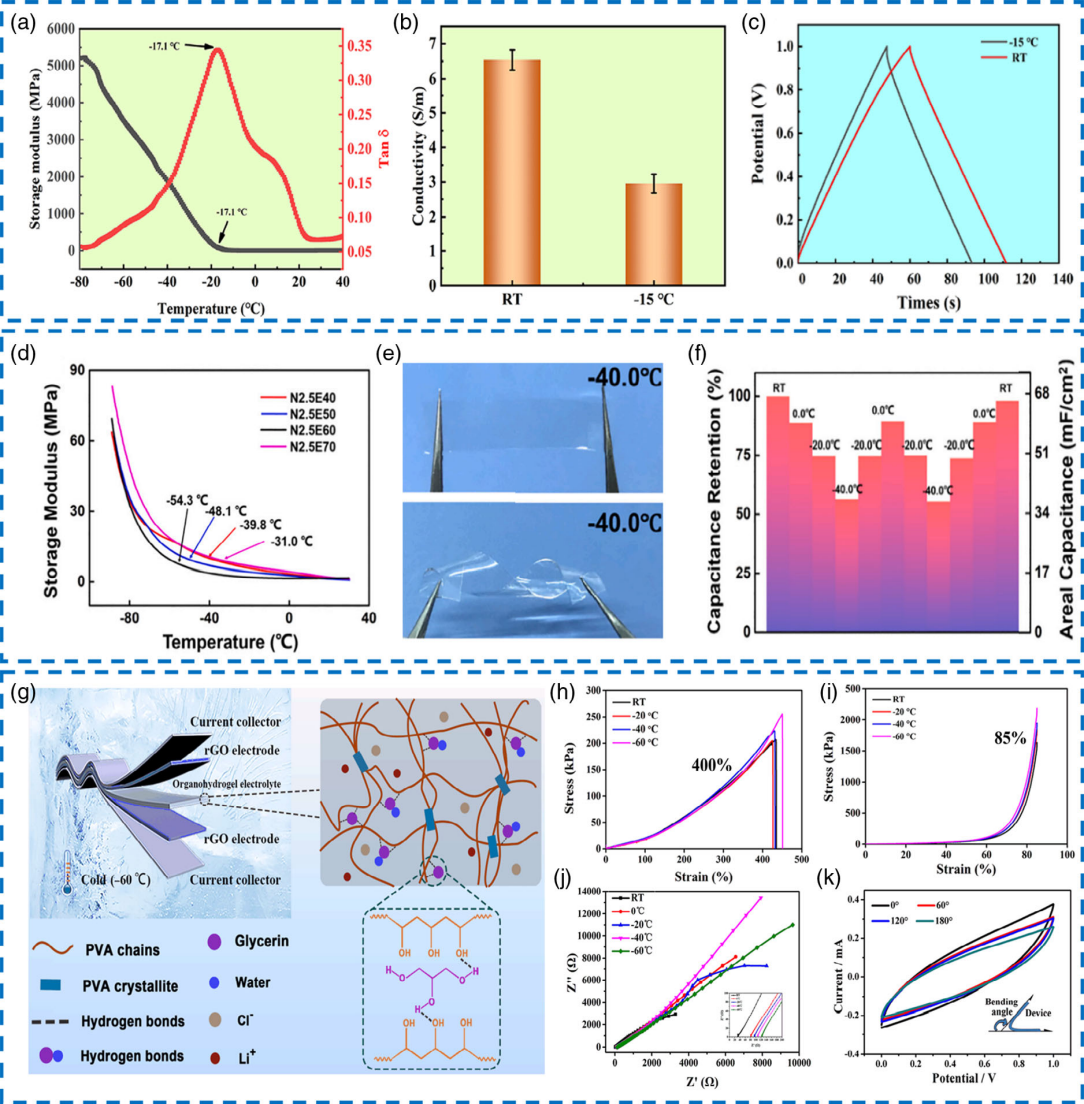
a) Dynamic thermomechanical analysis (DMA) curves of the PVA/SA/NaCl/PEG/H_2_O organo‐hydrogel electrolyte. b) Ionic conductivities of this organo‐hydrogel electrolyte at –15 °C and room temperature. c) Comparison of GCD curves at 2 mA cm^−1^ under different temperatures. a–c) Reproduced with permission.^[^ ^103^ ^]^ Copyright 2021, American Chemical Society. d) Storage modulus of the cellulose‐based organo‐hydrogel electrolyte as a function of temperature. e) Photograph of mechanical stability at –40 °C. f) Continuous changes in capacitance performance during the repeated cooling–heating cycles. d–f) Reproduced with permission.^[^ ^56^ ^]^ Copyright 2021, Elsevier. g) Schematic diagram of the PVA‐based organo‐hydrogel electrolyte applied to the anti‐freezing supercapacitor. h) Tensile *σ*–*ε* curves, i) compressive *σ*–*ε* curves, and j) electrochemical impedance spectroscopy (EIS) of this organo‐hydrogel electrolyte under different temperatures. k) Cyclic voltammetry (CV) curves of the supercapacitor under different bending angles. g–k) Reproduced with permission.^[^ ^104^ ^]^ Copyright 2021, American Chemical Society.

To achieve the above‐mentioned two ideas, an anti‐freezing organo‐hydrogel electrolyte was obtained through dispersing PVA and SA into PEG/water mixed solvent medium followed by freezing−thawing and soaking treatments.^[^ ^103^ ^]^ It should be mentioned that each basic unit of PEG bound at least two water molecules due to the presence of abundant hydrophilic functional groups. As a natural polysaccharide, SA exhibited ideal ionic hydration by the right of many carboxylic acid ions (–COO^−^), considering both ionic conductivity and inherent flexibility at low temperatures is imperative. Dynamic thermomechanical analysis (DMA) demonstrated that the PVA/SA/NaCl/PEG/water organo‐hydrogel electrolyte transformed from the elastic state to the glass state when the temperature was gradually adjusted to −17.1 °C, indicating the effective reduction of the freezing point (**Figure** **8**a). Figure 8b displayed that the immersion of saturated NaCl aqueous solution and the restriction of free water activity allowed high ionic conductivity of 2.95 ± 0.27 S m^−1^ to be maintained at –15 °C. Unexpectedly, the organo‐hydrogel showed a higher tensile strength of 1.61 ± 0.04 MPa at −15 °C than that at room temperature (1.43 ± 0.06 MPa), which may be the enhanced crystallinity of PVA after freezing. Accordingly, the final flexible supercapacitor delivered quasi‐rectangular CV curves and symmetric GCD profiles at −15 °C, and nearly 88.3% of capacitance retention (91.5 mF cm^−2^) relative to room temperature (103.6 mF cm^−2^) could be realized (Figure 8c).

Using natural substances to replace non‐degradable synthetic polymers is environmentally friendly and sustainable. Qin et al. immersed the crosslinked cellulose network into NaCl/EG/H_2_O solution to form a green organo‐hydrogel electrolyte, where the solvation state of macromolecular chains was adjusted and intra‐/inter‐molecular interactions were changed.^[^ ^56^ ^]^ The ternary components, including salt, organic compound, and water, could be coagulants for cellulose regeneration. Besides, the self‐aggregation and self‐association of cellulose chains were affected by the interactions among ion–cellulose–solvent systems, which would play a significant role in the final structure and functions of cellulose‐based organo‐hydrogel electrolytes. It was worth noting that the low‐temperature resistance of the electrolyte was closely related to the EG content. With the increase of EG content, the freezing point first declined and then elevated. The lowest freezing point was –54.3 °C at 60% of EG content (Figure 8d). Benefiting from the improved anti‐freezing property, the cellulose‐based organo‐hydrogel electrolyte was twisted without destruction at −40 °C (Figure 8e). As a result, the CV curves of the supercapacitor with the function of frost resistance were close to a rectangle shape at −40 °C, implying good capacitive behavior. It held 55% initial capacitance when cooled to −40 °C (Figure 8f). Both CV areas and ionic conductivity are slightly reduced, which may be attributed to the sluggish diffusion kinetics at subzero temperatures.

Till now, the lowest reported temperature for the supercapacitor based on the organo‐hydrogel electrolyte to normally operate is −65 °C.^[^ ^32^ ^]^ The presence of DMSO enabled the PVA/graphene organo‐hydrogel electrolyte to be curled easily and maintain a relatively excellent ionic conductivity value of 1.0 S m^−1^ at the extremely low temperature of −65 °C. Of particular note, the supercapacitor still showed a specific capacitance of up to 225 F g^−1^ under the aforementioned harsh temperature condition, which was 93% of the capacitance at room temperature. For the practical application, the supercapacitor, which was folded 10 times, successfully lit an LED at ultra‐low temperatures. In another case, an organo‐hydrogel composed of PVA, LiCl, and Gly/water solution was also stable at a relatively low temperature of −60 °C (Figure 8g)^[^ ^104^ ^]^ Regarding the low‐temperature mechanical tests, the prepared organo‐hydrogel electrolyte was stretched to 400% and compressed by 85% without fracture (Figure 8h,i). The storage modulus (G’) also delivered a steady upward trend during the gradual cooling process, which validated its stable mechanical properties. For the electrochemical performance, a high ionic conductivity of 1.6 S m^−1^ at −60 °C was retained (Figure 8j). At this temperature, the flexible supercapacitor equipped with the organo‐hydrogel electrolyte could still be bent under different angles without solidifying the aqueous electrolyte (Figure 8k) and exhibited a decent specific capacitance of 7.73 F g^−1^ at 2 mV s^−1^.

## 4 Organo‐Hydrogel Electrolytes for Zn‐Based Batteries

Metallic Zn is a prospective alternative to battery materials due to its abundant reserves, low redox potential, and satisfactory stability, making it reasonable for application in next‐generation portable energy storage systems.^[^ 105, 106, 107 ^]^ Considerable progress have been invested in Zn‐based aqueous batteries, including Zn‐ion and Zn–air batteries. Their solid‐state counterparts have undergone relatively few practical applications, especially extreme environmental ordeals. Additionally, the water escape in the aqueous electrolyte and the parasitic reactions in the Zn anode both cause the inherent issues of short‐circuit and self‐discharge, which limit the further development of such batteries.^[^ 108, 109 ^]^ Fortunately, developing organo‐hydrogel electrolytes has been reported as an effective protocol in alleviating the above problems. Due to the distinct quasi‐solid‐state properties of organo‐hydrogels, salt ions and solvent molecules in the gel matrix can be stably dispersed and transmitted, avoiding interference from the external environment.^[^ ^110^ ^]^ Moreover, soft and viscous organo‐hydrogel electrolytes in flexible Zn‐based batteries show intimate contact with electrodes to reduce the interfacial resistance and are able to suppress Zn dendritic growth to a certain extent by their intrinsic mechanical strength.^[^ ^111^ ^]^

### 4.1 Flexible Organo‐Hydrogel Electrolytes for Zn‐Ion Batteries

Apart from developing organo‐hydrogel electrolytes with superior intrinsic properties, the compatibility between electrolyte and electrode also should be paid attention to prolong the durability of flexible Zn‐ion batteries.^[^ ^112^ ^]^ Crucially, the materials used for the cathode, electrolytes, and Zn anode are different, which will put high demands on interface stability. It is meaningful to fabricate a multifunctional organo‐hydrogel electrolyte to achieve the all‐around regulation of electrode reactions. For instance, a functional organo‐hydrogel electrolyte containing PAM, ZnSO_4_, Gly, and AN was prepared, which offered a comprehensive combination of several advantages, including satisfactory mechanical properties, excellent ionic conductivity, and tunable bonding interaction.^[^ ^113^ ^]^ DFT and FTIR demonstrated that water molecules tended to interact with oxygen‐containing groups in gel matrix rather than Zn^2+^ ions, thus resulting in reversible Zn striping/plating behavior, high stability, and broad temperature adaptability (–20 to 60 °C) in Zn‐based energy storage. The Zn–V_2_O_5_ batteries using this organo‐hydrogel electrolyte showed outstanding cycling stability (Zn||Zn symmetric cell stably cycles more than 3000 h), superior reversibility (Coulombic efficiency approaches 99.5%) and improved capacity performance (185 mAh g^−1^ at the current density of 5 A g^−1^ for 10 000 cycles). Moreover, these batteries still exhibited commendable cyclic stability (Zn||Zn symmetric cell steadily cycles over 500 h) and excellent capacity at −20 °C or 60 °C.

**Figure 9:**
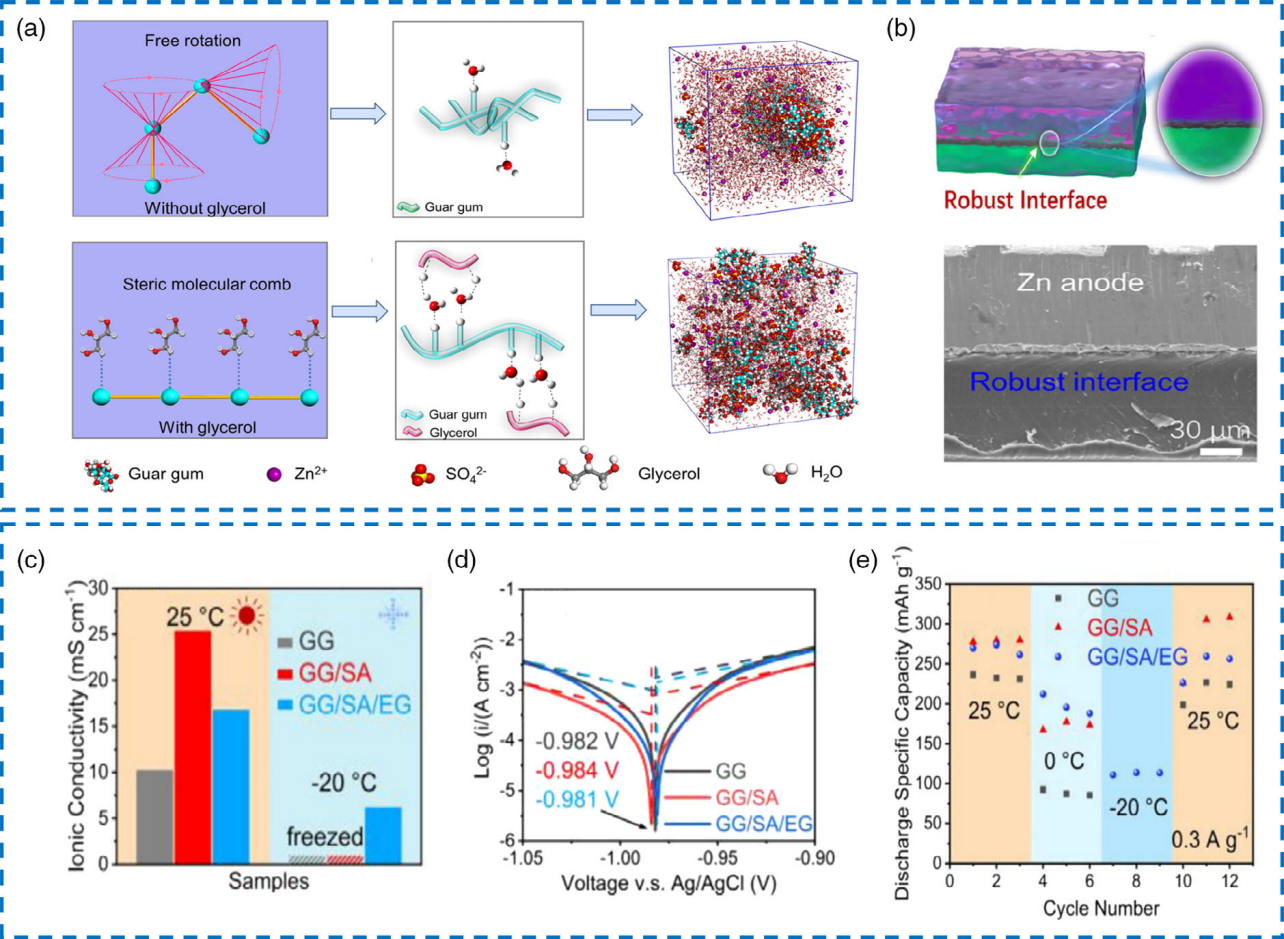
a) Illustration of the steric molecular combing effect. b) Scanning electron microscopy (SEM) image of the compact Zn anode interface after 250 cycles in a guar‐gum‐based organo‐hydrogel electrolyte. a,b) Reproduced with permission.^[^ ^114^ ^]^ Copyright 2022, American Chemical Society. c) Comparison of the ionic conductivity. d) Tafel plots of different organo‐hydrogels. e) Discharge capacities of Zn‐ion batteries using different organo‐hydrogel electrolytes under different temperatures. c,d) Reproduced with permission.^[^ ^115^ ^]^ Copyright 2021, Elsevier.

To better address the possible damage of electrode–electrolyte interface during long‐term operation, Liu et al. reported a novel steric molecular combing effect to prepare an electrolyte based on guar gum (GG) with ultrafast self‐healing capacity and dynamically adaptive capability.^[^ ^114^ ^]^ Molecular dynamics illustrated that the spontaneous free rotation of the C–C single bond from the GG in the ZnSO_4_ solution would result in irregular curling. Coiled macromolecules usually behave as aggregates due to the enhanced hydrogen bonding interaction. Then, shielding of active hydroxyl groups in the backbone made of GG‐based electrolyte is less capable of self‐healing. In stark contrast, after Gly was introduced into the solvent to form a new hydrogen bond system, a special steric molecular comb was thus constructed, which would adjust the molecular configuration of GG from a coiled state to a straight chain state and expose more active hydroxyl groups for boron–ether crosslinking (**Figure** **9**a). The effective transformation of molecular structure also endowed the organo‐hydrogel electrolyte with superfast self‐healing capability. The simulated needle pierces experiment proved that the Gly‐modified GG‐based organo‐hydrogel electrolyte recovered quickly after the shrinkage of Zn dendrites, indicating the formation of a dynamic self‐adaptive electrode‐electrolyte interface. Besides, the robust interfacial contact during repeated Zn stripping/plating process was visually characterized by SEM (Figure 9b). Consequently, the Zn||Zn symmetrical cell in the GG/ZnSO_4_/Gly/water organo‐hydrogel electrolyte system exhibited a polarization overpotential of about 50 mV and a superior life span of 1500 h at 1 mA cm^−2^ and 1 mAh cm^−2^, evidently longer than that of the GG/ZnSO_4_/water hydrogel electrolyte system. Similarly, the full Zn‐MnO_2_ battery with the self‐healing organo‐hydrogel electrolyte maintained a high specific capacity of 200 mAh g^−1^ (95% of capacity retention) after 500 cycles, again better than that of quasi‐solid‐state electrolyte without Gly. Concerning another GG‐based organo‐hydrogel electrolyte, Wang et al. blended SA into the system to construct a crosslinked composite‐gel network with better ionic conductivity and flexibility.^[^ ^115^ ^]^ The highest ionic conductivity value reached 16.81 mS cm^−1^ (Figure 9c). Besides, mechanical strength and frost resistance were improved by a solvent‐displacement‐induced strategy using EG. For the electrochemical stability test of the Zn anode, the GG/SA organo‐hydrogel electrolyte with a corrosion current of 0.2939 mA cm^−2^ and a corrosion potential of −0.981 V showed less corrosive harm on Zn foil compared with the corresponding GG hydrogel electrolyte (Figure 9d). In addition, the Zn||Zn symmetric cell at 25 °C or –20 °C showed an excellent cyclic performance exceeding 200 h with no significant difference in polarization voltage at the current density of 0.2 mA cm^−2^. Further, as shown in Figure 9e, the Zn‐MnO_2_ full battery with GG/SA organo‐hydrogel electrolyte also delivered the highest electrochemical performance (25 °C: 354.9 mAh g^−1^ at 0.15 A g^−1^, 91.52% of capacity retention after 1000 cycles, –20 °C: 181.5 mAh g^−1^ at 0.1 A g^−1^, 80.39% of capacity retention after 100 cycles) compared with parallel samples.

**Figure 10:**
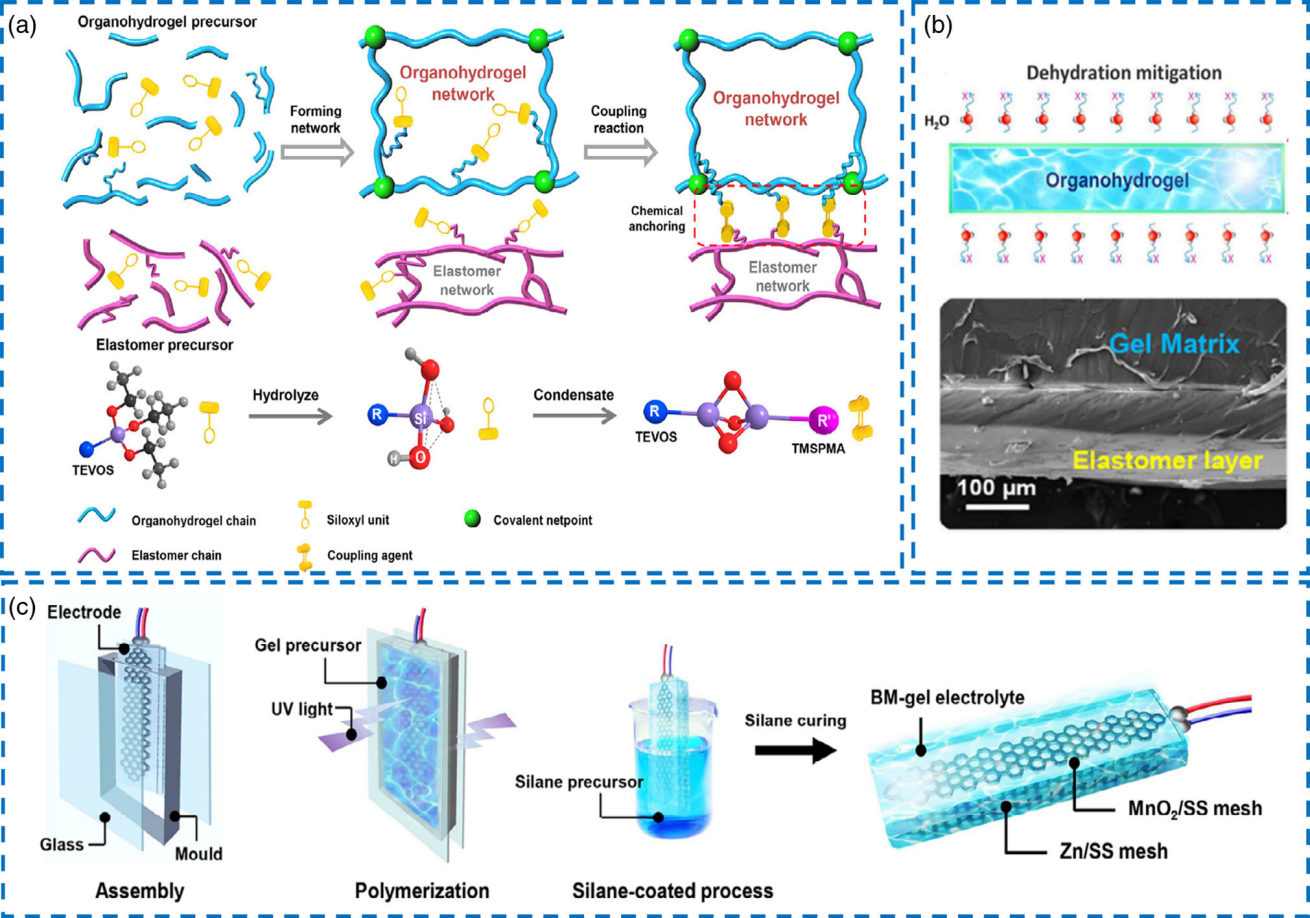
a) Illustration of the bonding between the organo‐hydrogel electrolyte and the elastomer layer. b) SEM image of the elastomer‐coated organo‐hydrogel electrolyte. c) Schematic illustration of the construction process of solid‐state Zn‐MnO_2_ battery. a–c) Reproduced with permission.^[^ ^116^ ^]^ Copyright 2019, Wiley‐VCH.

The stability of aqueous Zn‐ion batteries is mainly affected by the swelling of absorbing external water and the dehydration of internal evaporation. To solve this obstacle, Mo et al. reported an elastomer‐coated EG‐based Zn‐alginate/PAM organo‐hydrogel (BM‐gel) electrolyte with superior moisture lock‐in and anti‐freezing properties inspired by the operation mechanism of epidermal tissue.^[^ ^116^ ^]^ The gel matrix was crosslinked by PAM, triethoxy(vinyl)silane (TEOVS), and elastomer‐poly(dimethylsiloxane) (PDMS) in three sequential steps, where the silane coupling agent played an essential role in the condensation and bonding process (**Figure** **10**a). As for the inner structure, cross‐sectional SEM directly presented the conformal and tight interface between the superficial coating and organo‐hydrogel network (Figure 10b). In the dehydration test, the BM‐gel was placed in the environment with a temperature of 25 °C and a humidity of 50%. Impressively, the weight of the BM‐gel electrolyte changed only slightly (<2%) after 30 days, whereas the unmodified PAM‐hydrogel electrolyte showed a rapid mass loss in the initial 5 days and eventually became a dry scaffold. The same results were also obtained at either low or high temperatures. Besides, the dye impregnation experiment also proved that the elastomer coating delayed material exchange between the inner gel and the external environment, which was conducive to long‐term electrolyte stability. Under the regulation of EG, BM‐gel electrolytes exhibited better tensile and compressive strength in normal, cold, and hot conditions. The ionic conductivity also did not fluctuate significantly with temperature change. Therefore, the constructed rechargeable Zn–MnO_2_ battery displayed remarkable electrochemical performance at whole temperatures (Figure 10c), including over 70% of specific capacity and approximately 100% Coulombic efficiency. Moreover, the batteries maintained high capacity without appreciable deterioration under different destructive treatments.

### 4.2 Flexible Organo‐Hydrogel Electrolytes for Zn‐Air Batteries

As a mature energy storage technology with a century‐old history, Zn–air batteries are perceived as the most promising alternative to commercial lithium‐ion batteries, as the energy density of the former is about five times that of the latter.^[^ 117, 118, 119 ^]^ The development of Zn–air batteries is constantly towards the breakthrough of flexibility and environmental adaptability. The currently reported flexible Zn–air batteries are primarily divided into three configurations: cable‐type,^[^ ^120^ ^]^ sandwich‐type,^[^ ^121^ ^]^ and in‐plane‐type.^[^ ^122^ ^]^ In terms of electrolyte composition, KOH is often required to be added since its corresponding aqueous solution exhibits low viscosity, high ionic conductivity (over 50 S m^−1^ at room temperature), and excellent oxygen diffusion coefficient for achieving prominent electrochemical reaction kinetics and mass transfer during the long‐term charging/discharging process.^[^ ^123^ ^]^ However, the inferior water retention of those conventional PVA‐based hydrogel electrolytes generally triggers rapid attenuation in ionic diffusion and obvious deterioration in battery performance.^[^ ^124^ ^]^ In contrast, organo‐hydrogel electrolytes own a 3D crosslinked network, which can conserve solvents with different polarities and modulate solvated structures to balance ionic conductivities and mechanical properties. At the same time, systematic improvement of portability and solvent protection in organo‐hydrogel electrolytes can significantly impact the energy density, power output, and cycle stability of the final Zn–air batteries.

**Figure 11:**
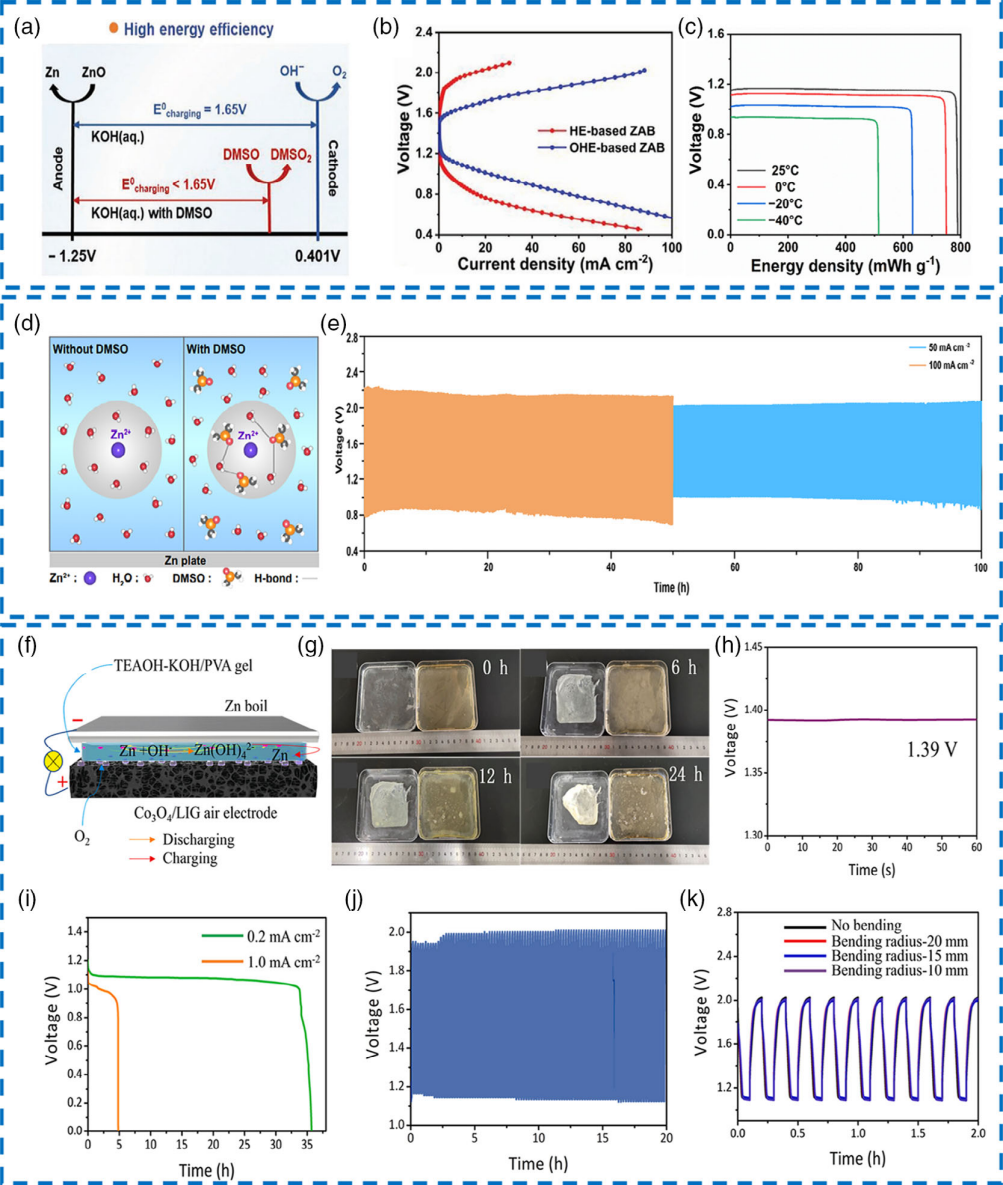
a) The oxidation reaction potential for the air cathode in the presence of DMSO. b) Charge/discharge profiles of the flexible Zn–air batteries using different electrolytes. c) Specific discharge capacity plots obtained at 2 mA cm^−2^ under different temperatures. a–c) Reproduced with permission.^[^ ^127^ ^]^ Copyright 2022, Wiley‐VCH. d) Zn^2+^ solvation structure under the regulation of dimethyl sulfoxide (DMSO) and H_2_O molecules. e) Charging/discharging performance of the flexible Zn–air batteries at 50 and 100 mA cm^−2^. d,e) Reproduced with permission.^[^ ^31^ ^]^ Copyright 2022, Springer Nature. f) Component of the Zn–air battery using TEAOH/PVA‐based organo‐hydrogel electrolyte. g) Water retention test of the organo‐hydrogel. h) Open‐circuit potential curve. i) Galvanostatic discharge profiles. j) Cyclic galvanostatic discharge/charge profiles at 0.5 mA cm^−2^ for up to 20 h. k) Galvanostatic discharge/charge profiles at 0.5 mA cm^−2^ for up to 2 h under different deformation conditions. f–k) Reproduced with permission.^[^ ^129^ ^]^ Copyright 2022, Elsevier.

DMSO possesses a high donor number (29.8), good chemical stability, and favorable polarity in alkaline media, forming effective hydrogen bonds with water molecules through S=O. More importantly, DMSO is able to dissolve many Zn‐based conductive salts, ensuring reversible Zn dissolution/deposition and reducing detrimental Zn surface passivation.^[^ 125, 126 ^]^ Therefore, considering its strong binding with Zn^2+^ ions and solvent molecules, DMSO‐based mixed solvent systems have been widely applied in functional organo‐hydrogel electrolytes for flexible Zn–air batteries. For instance, Jiang et al. prepared an anti‐freezing organo‐hydrogel electrolyte by soaking PAMPS/PAM hydrogel in 5 M KOH aqueous electrolyte with a DMSO additive.^[^ ^127^ ^]^ It is worth mentioning that DMSO plays a dual role in regulating the electrochemical reactions of electrolytes. On the one hand, DMSO restrained the ice crystal formation and improved the freezing resistance. DSC curves depicted that the crystallization peak of the DMSO‐based organo‐hydrogel electrolyte (–57.4 °C) was lower than those of the hydrogel electrolyte (–36.4 °C) and the original hydrogel (3.2 °C). At subzero temperatures, this organo‐hydrogel electrolyte remained transparent and elastic, and it still displayed high ionic conductivities (4.33 S m^−1^ at –20 °C and 1.72 S m^−1^ at –40 °C). Second, the oxidation potential of DMSO was lower than that of the oxygen evolution reaction (OER) potential of RuO_2_/Co_3_O_4_‐based air electrodes, which decreased the charging voltage and promoted energy efficiency (**Figure** **11**a). As illustrated in GCD profiles, the charging voltage and the voltage gap of flexible Zn–air battery using organo‐hydrogel electrolyte were lower than those of hydrogel electrolyte counterpart (Figure 11b). Correspondingly, a considerably high energy efficiency of 74.2% and a long cyclic lifespan of 177 cycles were successfully achieved. In addition, the optimized battery operated with a large specific capacity of 562 mAh g^−1^ and an excellent energy density of 523.4 Wh kg^−1^ at –40 °C (Figure 11c). In another similar case, the introduction of a DMSO additive into a PAM‐based hydrogel electrolyte was able to reconstruct the Zn^2+^ solvation sheath structure, thus inhibiting dendrite growth and hydrogen evolution reaction (HER) on the Zn anode (Figure 11d).^[^ ^31^ ^]^ The synergistic binding effect of alkalized amide groups and DMSO molecules on the water showed organo‐hydrogel superior thermal stability and freezing resistance. As a result, a robust cyclic stability of 50 h under a high current density of 100 mA cm^−2^ (Figure 11e) and a versatile temperature adaptability (−60 to 60 °C) for quasi‐sold‐state Zn–air batteries could be simultaneously realized.

As for other organic additives, Gly, a biodegradable and nontoxic resource, can affect the freezing and volatilization of electrolytes and be effectively compatible with electrode materials. Chen et al. prepared a PAM/PAA dual network organo‐hydrogel electrolyte to investigate the influence of Gly on the mechanical properties of gels and the electrochemical performances of final Zn–air batteries.^[^ ^128^ ^]^ With the elevation of Gly mass content from 0% to 70%, the strength and stretchability of the organo‐hydrogel increased firstly and then decreased. The largest stress strength and tensile strain were 145.5 kPa and 500%, respectively. However, excessive Gly was unfavorable to the dissolution of monomers (AA and AM), resulting in the decay of mechanical properties. In the low‐temperature and baking environment, the presence of Gly made the organo‐hydrogels remain transparent and scalable. The lap shear strength tests certified that Gly enhanced the adhesion by forming the multidentate alkoxide bonds and hydrogel bonds on the surface of metal electrodes from −20 to 70 °C. In this role, the assembled flexible Zn–air battery delivered the largest output power density of 11.8 mW cm^−2^, a maximum specific capacity of 663.25 mAh g^−1^ at 25 °C. Moreover, the capacity retentions of the batteries at different temperatures relative to the performance at 25 °C were 72.32% (466.44 mAh g^−1^, –20 °C), 92.13% (594.21 mAh g^−1^, 0 °C), and 75% (483.68 mAh g^−1^, 70 °C). In the actual application, the batteries continuously powered the timers under various outer conditions, including high or low temperatures, burning, hammering, cutting, and bending. Beyond that, tetraethyl ammonium hydroxide (TEAOH) as either the adhesive binder or the conductive component was added into the PVA‐based gel to construct a tight electrode/electrolyte/air three‐phased interface (Figure 11f).^[^ ^129^ ^]^ The moisturized state of the gel electrolyte was maintained for a long time (Figure 11g). TEA^+^ ions were also beneficial to elevate the deposition potential of Zn^2+^ ions, thereby increasing the output potential of the integrated thin‐film Zn–air battery with the laser‐sintering Co_3_O_4_/laser‐induced graphene (LIG) cathode (Figure 11h). The most prominent specific capacity obtained by normalizing to the consumed Zn mass was 712 mAh g^−1^ at 0.2 mA cm^−2^ (Figure 11i). Moreover, the aforementioned battery maintained a long life of 20 h with average charge and discharge voltages of 2.00 and 1.13 V, respectively. (Figure 11j). For the bending performance tests, Zn–air batteries showed steadily reversible GCD profiles without performance degradation while setting bending radii as 10, 15, and 20 mm, respectively (Figure 11k).

## 5 Summary and Perspectives

This article discusses the latest developments of several aqueous energy storage techniques with superior environmental adaptability using organo‐hydrogel electrolytes. As the “blood” of flexible devices, multifunctional organo‐hydrogel electrolytes prominently affect electrochemical performances under extreme environmental conditions through the modulation of ionic transfer, solvation structure, and interfacial interaction. Hence, the chemical constitution, synthesis methods, properties validation, and electrochemical application of organo‐hydrogel electrolytes are systematically summarized (**Table** **1**). To date, two main synthesis methods have been exploited for the organo‐hydrogels with mixed solvents, namely the “physical crosslinking strategy” and “chemical crosslinking strategy”. The solvent can be dispersed in the network by in situ crosslinking or solvent displacement. Among all organic solvents, excellent chemical and electrochemical stability of organic hydrogels requires the organic solvent with high donor numbers to form strong interactions. For example, Gly‐based organo‐hydrogels exhibit better freezing resistance relative to EG‐based organo‐hydrogels, which can be attributed to more hydrogen bonds constructed by three hydroxyl groups in Gly with surrounding water molecules. Besides, a high dielectric constant is also indispensable for the dissociation of ions to enhance the electrical conductivity of organo‐hydrogel electrolytes. A wide variety of energy storage devices, such as supercapacitors, Zn‐ion batteries, and Zn–air batteries, can be assembled with unique organo‐hydrogels through the addition of organic solvents and the modification of polymer chemistries. These devices are characterized by broad temperature tolerance, robust mechanical strength, and chemical stability.

**Table 1:** Various organo‐hydrogel electrolytes with different properties for flexible aqueous energy storage devices

No.	Organo‐hydrogel electrolytes	Content of organic solvents	Temperature adaptation	Mechanical properties	Ionic conductivity	Application	Cyclic stability	Reference
1	PVA/LiCl/EG/H_2_O	33 wt%	−40 °C to room temperature	fracture strain of 300%	≈2 S m^−1^	supercapacitor	88.3% capacitance retention (5000 cycles)	[26]
2	PAM/H_2_SO_4_/EG/H_2_O	50 vol%	−30 to 25 °C	tensile strain of 350%	5.33 S m^−1^	supercapacitor	84.7% capacitance retention (50 000 cycles, 100 mV s^−1^)	[27]
3	Poly(AA*‐co*‐AM)/AP/KCl/PEG/H_2_O	1.87 wt%	−20 to 80 °C	fracture strain/stress of 1089%/200 kPa	≈2 S m^−1^	supercapacitor		[28]
4	PAMPS/PAM/LiCl/EG/H_2_O	50 mol%	−20 to 80 °C	fracture strain/stress of 390%/0.84 MPa	2.29 S m^−1^	supercapacitor	91.3% capacitance retention (10 000 cycles)	[29]
5	PVA/PAMAA/NaCl/Gly/H_2_O	43 wt%	−20 °C to room temperature	fracture strain/stress of 1330%/223 kPa	>1.2 S m^−1^	supercapacitor	90.2% capacitance retention (5000 cycles, 5 mA cm^−2^)	[30]
6	PVA/graphene/H_2_SO_4_/DMSO/H_2_O	67 mol%	−65 to 25 °C	fracture strain/stress of 500%/2.58 kPa	2.67 S m^−1^	supercapacitor	83.6% capacitance retention (2000 cycles)	[33]
7	P(AM*‐co*‐DMAEMA)‐AMP/gelatin/LiCl/Gly/H_2_O	33 vol%	−40 to 25 °C	fracture strain/stress of 2301%/151.8 kPa	1.36 S m^−1^	supercapacitor	86.4% capacitance retention (10 000 cycles)	[35]
8	Gelatin/PAA/NaCl/Gly/H_2_O	40 vol%	−20 °C to room temperature	fracture strain/stress of 580%/0.46 MPa	>0.7 S m^−1^	supercapacitor	94% capacitance retention (2000 cycles)	[42]
9	PVA/ARS/H_2_SO_4_/EG/H_2_O	60 wt%	−37 °C to room temperature	tensile strain of 330%	2.52 S m^−1^	supercapacitor	91% capacitance retention (5000 cycles)	[45]
10	Cellulose/NaCl/EG/H_2_O	60 vol%	−54.3 °C to room temperature	tensile strain of 242%	1.92 S m^−1^	supercapacitor	80% capacitance retention (1000 cycles)	[56]
11	PAMPS/PAM/ZnCl_2_/NH_4_Cl/EG/H_2_O	90 vol%	−30 to 120 °C	fracture strain/stress of 166%/0.22 MPa	>0.35 S m^−1^	supercapacitor	90.2% capacitance retention (100 000 cycles, 2 A g^−1^)	[57]
12	PVA/PAMAA/CaCl_2_/DMSO/H_2_O	50 vol%	room temperature	fracture strain/stress of ≈ 1500%/270 kPa	2.24 S m^−1^	supercapacitor	75% capacitance retention (3000 cycles, 5 mA cm^−2^)	[58]
13	Starch/PVA/CaCl_2_/Gly/H_2_O	40 wt%	−20 °C to room temperature	fracture strain/stress of 793%/0.53 MPa	1.0 S m^−1^	supercapacitor	84.5% capacitance retention (8000 cycles, 5 mA cm^−2^)	[62]
14	PVA/CMC/LC/KOH/EG/H_2_O	67 vol%	−40 to 60 °C	fracture strain/stress of 562%/0.1 MPa	8.3 S m^−1^	supercapacitor	75% capacitance retention (10 000 cycles, 3 A g^−1^)	[85]
15	PAMPs*‐co*‐PAAM/PVA/LiCl/EG/H_2_O	50 vol%	−20 to 80 °C	fracture strain/stress of 648%/75.5 kPa	2.67 S m^−1^	supercapacitor	88% capacitance retention (10 000 cycles, 2 A g^−1^)	[97]
16	PU/NaClO_4_/ACN/H_2_O	61.6 mol%	room temperature	fracture strain/stress of 2220%/4.5 MPa	1.95 S m^−1^	supercapacitor	95.2% capacitance retention (10 000 cycles, 5 A g^−1^)	[100]
17	PVA/SA/NaCl/PEG/H_2_O	14.3 wt%	−15 °C to room temperature	fracture strain/stress of 439%/1.43 MPa	6.54 S m^−1^	supercapacitor	81.9% capacitance retention (8000 cycles, 5 mA cm^−2^)	[103]
18	PVA/LiCl/Gly/H_2_O	33 vol%	−60 °C to room temperature	lap shear strength of 14.1 kPa	14 S m^−1^	supercapacitor	100% capacitance retention (1000 cycles, 0.2 A g^−1^)	[104]
19	EG‐waPUA/PAM/ZnSO_4_/MnSO_4_/H_2_O	24 wt%	−20 to 20 °C	fracture strain/stress of 1100%/85 kPa	1.68 S m^−1^	Zn‐ion battery	88.6% capacitance retention (600 cycles, 0.2 A g^−1^)	[31]
20	AGr/ZnSO_4_/PEG/H_2_O	6 wt%	20 to 100 °C		5.16 S m^−1^	Zn‐ion battery	70.2% capacitance retention (500 cycles, 1 A g^−1^)	[34]
21	Cellulose/TEOS/ZnSO_4_/MnSO_4_/Gly/H_2_O	23 vol%	−40 to 60 °C	fracture strain/stress of 846.5%/2.11 kPa	3.23 S m^−1^	Zn‐ion battery	99.2% capacitance retention (2000 cycles, 3 A g^−1^)	[75]
22	PVA/borax/ZnSO_4_/MnSO_4_/Gly/H_2_O	17 wt%	−35 to 25 °C	fracture strain/stress of 502%/115.5 kPa	2.96 S m^−1^	Zn‐ion battery	93.7% capacitance retention (2000 cycles, 1 A g^−1^)	[110]
23	PAMPS/PAM/ZnSO_4_/DMSO/H_2_O	–	−20 °C to room temperature	fracture strain/stress of 2700%/103 kPa	2.16 S m^−1^	Zn‐ion battery	96% capacitance retention (1000 cycles, 3 A g^−1^)	[111]
24	PAM/ZnSO_4_/Gly/AN/H_2_O	20 vol%	−20 to 60 °C	be well stretched, twisted, compressed	1.394 S m^−1^	Zn‐ion battery	89% capacitance retention (10 000 cycles, 5 A g^−1^)	[113]
25	Guar gum/ZnSO_4_/Gly/H_2_O	9 vol%	room temperature	fast self‐healing		Zn‐ion battery	98.5% capacitance retention (10 000 cycles, 10 A g^−1^)	[114]
26	Guar gum/SA/ZnSO_4_/MnSO_4_/EG/H_2_O	30 vol%	−20 to 25 °C	be well stretched and twisted	2.537 S m^−1^	Zn‐ion battery	91.52% capacitance retention (1000 cycles, 6 A g^−1^)	[115]
27	Alginate/PAM/silane/ZnSO_4_/MnSO_4_/EG/H_2_O	30 vol%	−20 to 80 °C	tensile stress of about 50 kPa	1.63 S m^−1^	Zn‐ion battery	81% capacitance retention (500 cycles, 1.6 A g^−1^)	[116]
28	PAM/Zn(CH_3_COO)_2_/KOH/DMSO/H_2_O	33 mol%	−60 to 60 °C		0.26 S m^−1^	Zn‐air battery	100 h at 50 mA cm^−2^	[32]
29	PAMPS/PAM/KOH/DMSO/H_2_O	3 wt%	−40 to 25 °C	fracture strain/stress of 220%/206 kPa	8 S m^−1^	Zn‐air battery	59 h (177 cycles) at 1 mA cm^−2^	[127]
30	PAM/PAA/Zn(CH_3_COO)_2_/KOH/Gly/H_2_O	50 wt%	−20 to 70 °C	fracture strain/stress of 500%/5.04 MPa	0.45 S m^−1^	Zn‐air battery	10 h (100 cycles) at 1 mA cm^−2^	[128]
31	PVA/TEAOH/KOH/H_2_O	40 vol%	room temperature			Zn‐air battery	20 h at 0.5 mA cm^−2^	[129]

**Figure 12:**
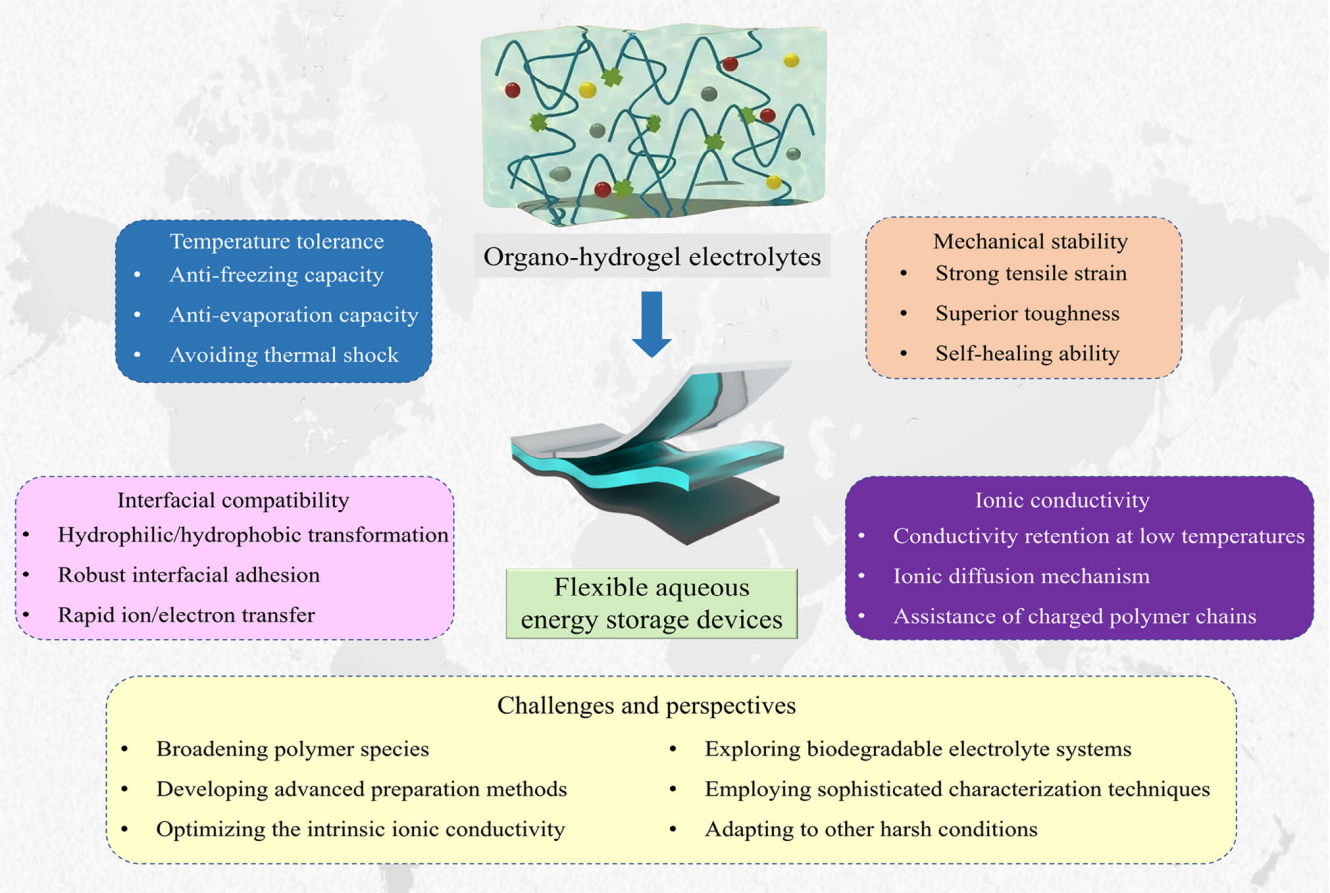
Summary and prospects of organo‐hydrogel electrolytes toward future flexible aqueous energy storage devices.

As a pivotal component in the quasi‐solid‐state device, the organo‐hydrogel electrolyte is associated with many critical issues in the energy storage device, including electrochemical performances, ionic conductivity, electrode/electrolyte interfaces, and flexibility. This would offer the materials more degree of freedom compared with the common hydrogel electrolyte. For instance, the organo‐hydrogel could be well viscose, which may optimize the interfacial interaction between electrode and electrolyte materials when being subjected to external mechanical deformations. Furthermore, the hydrogel electrolyte is usually fragile and rigid at subzero conditions. In contrast, the utilization of organo‐hydrogel materials benefits the water retention ability, in which the solvent water molecules could hardly evaporate and the relevant performances are well maintained. It is favorable to use organo‐hydrogel materials as electrolytes for flexible energy storage devices to meet different harsh test conditions. However, the primary research and commercialization of flexible organo‐hydrogel electrolytes still require a lot of effort. Based on the above analyses, some challenges and perspectives are coming up, looking forward to promoting the development of organo‐hydrogel electrolytes beyond the laboratory scale (**Figure** **12**). 1) At present, the polymer skeleton materials of organo‐hydrogels are relatively limited, mainly restricted by the solubility of polymers in mixed solvents with different polarities. Different functional groups, such as halogen atoms, can be attached to commonly used polymer chains to realize several fascinating inherent properties. For instance, F‐containing polymers can be stabilized in strong acid and alkali solutions owing to the high electronegativity of F.^[^ ^130^ ^]^ Such wide pH adaptation is expected to endow the flexible Zn–air batteries with stable operation in the entire pH environment. At the same time, the rapid development of machine learning and high‐throughput experiments can also help to screen potential candidate polymer materials and organic solvents from massive material databases. 2) The last large‐scale promotion has to enrich the existing preparation methods of organo‐hydrogel electrolytes. Further optimizations in inorganic fillers, multiple hybrid gel networks (interpenetrating frameworks), doping, and manufacturing processes are needed to bring the organo‐hydrogel electrolytes into future practical flexible energy storage devices. Luckily, some state‐of‐art technologies like 3D printing have made significant progress in the miniaturization and patterning of hydrogel‐based devices.^[^ ^131^ ^]^ These successful experiences can also inspire the advanced preparation of organo‐hydrogel electrolytes. 3) Compared with conventional hydrogel electrolytes, low ionic conductivity at room temperature is an inherent defect of organo‐hydrogel electrolytes. Optimizing ionic conductivity must consider the used conductive salts, while more detailed works and theoretical calculations are also required in this field. It can be observed that conductive ions with different valence states and sizes will have different interactions with polymer chains. For instance, Na_2_SO_4_ and ZnCl_2_ can be smoothly along the PVA chains. However, PVA will be flocculated once Zn^2+^ ions and SO_4_^2−^ ions coexist.^[^ ^8^ ^]^ In addition, the molecular weight, side group length, crosslinking degree, and charged properties of polymers could be modified to affect the transport rate of ions. Zwitterionic gels have been reported to possess special ion migration channels, which can be further applied in organo‐hydrogel electrolytes to improve ionic conductivity. 4) Environmental factors should be considered when exploring synthetic processes and electrolyte materials to meet the requirements of sustainable social development. Although energy storage devices are rechargeable, they still face degradation problems after performance exhaustion. Current research should focus on developing naturally derived biomaterials, such as biodegradable synthetic polymers, biocompatible solvents, and conductive salts.^[^ ^132^ ^]^ However, some natural materials (cellulose, alginate, etc.) used in organo‐hydrogels, extra additives, or strong acids/alkalis are still required to stabilize these electrolyte systems. 5) The exploration of the electrochemical reaction mechanism is highly complex due to the multiple components inside the organo‐hydrogels. It is imperative to employ more advanced and accurate characterization techniques to reveal the ion transport and interface evolution during charging and discharging processes in different environments, such as cryo‐SEM and cryo‐STEM.^[^ ^133^ ^]^ Also, the highly quantitative and non‐destructive characterization of quasi‐solid‐state electrolytes under different temperatures or external stresses relies on currently popular in‐situ detection techniques, such as infrared spectroscopy, Raman spectroscopy, etc. Nevertheless, the construction of in‐situ analysis devices on existing flexible supercapacitors/batteries also faces huge technical bottlenecks. Furthermore, the coupling of electrochemical measurement platforms, stress application/temperature modulation systems, and real‐time detection devices should be comprehensively planned. 6) In addition to the harsh environments discussed in this review, some other particular scenarios may also need to be considered. For example, in an environment with high content of CO_2_, the carbonation reaction often occurs in the conventional electrolytes of alkaline Zn–air batteries. Thus, exploring a novel anti‐CO_2_ organo‐hydrogel electrolyte may become a new research hotspot. Pressure may also be an essential factor affecting the electrochemical performances of electrolytes in possible regions of plateaus or abyssal. Besides, in the underwater environment, the outer salt concentrations may interfere with the routine work of organo‐hydrogel electrolytes through the osmotic pressure.^[^ ^16^ ^]^ Therefore, targeted/systematic strategies to cope with the various extreme conditions may arise further consideration.

## Conflict of Interest

The authors declare no conflict of interest.

## References

[1] J. F. Wen , B. G. Xu , Y. Y. Gao , M. Q. Li , H. Fu , Energy Storage Mater. 2021, 37, 94.

[2] A. Sumboja , J. W. Liu , W. G. Zheng , Y. Zong , H. Zhang , Z. L. Liu , Chem. Soc. Rev. 2018, 47, 5919. 29947399 10.1039/c8cs00237a

[3] M. Parrilla , K. De Wael , Adv. Funct. Mater. 2021, 31, 2107042.

[4] S. Y. Lyu , H. J. Chang , F. Fu , L. Hu , J. D. Huang , S. Q. Wang , J. Power Sources 2016, 327, 438.

[5] S. Siddiqui , D. I. Kim , E. Roh , L. T. Duy , T. Q. Trung , M. T. Nguyen , N. E. Lee , Nano Energy 2016, 30, 434.

[6] X. L. Cheng , J. Pan , Y. Zhao , M. Liao , H. S. Peng , Adv. Energy Mater. 2018, 8, 1702184.

[7] G. Lee , D. Kim , D. Kim , S. Oh , J. Yun , J. Kim , S. S. Lee , J. S. Ha , Energy Environ. Sci. 2015, 8, 1764.

[8] Z. F. Wang , H. F. Li , Z. J. Tang , Z. X. Liu , Z. H. Ruan , L. T. Ma , Q. Yang , D. H. Wang , C. Y. Zhi , Adv. Funct. Mater. 2018, 28, 1804560.

[9] C. Y. Chan , Z. Wang , H. Jia , P. F. Ng , L. Chow , B. Fei , J. Mater. Chem. A 2021, 9, 2043.

[10] W. Li , J. Zhang , J. R. Niu , X. Jin , X. M. Qian , C. F. Xiao , W. Y. Wang , Nano Energy 2022, 99, 107359.

[11] C. Li , G. Q. Shi , Adv. Mater. 2014, 26, 3992. 24659376 10.1002/adma.201306104

[12] K. G. Cho , H. S. Kim , S. S. Jang , H. Kyung , M. S. Kang , K. H. Lee , W. C. Yoo , Adv. Funct. Mater. 2020, 30, 2002053.

[13] H. H. Rana , J. H. Park , E. Ducrot , H. Park , M. Kota , T. H. Han , J. Y. Lee , J. Kim , J. H. Kim , P. Howlett , M. Forsyth , D. MacFarlane , H. S. Park , Energy Storage Mater. 2019, 19, 197.

[14] A. Vioux , B. Coasne , Adv. Energy Mater. 2017, 7, 1700883.

[15] H. Peng , X. J. Gao , K. J. Sun , X. Xie , G. F. Ma , X. Z. Zhou , Z. Q. Lei , Chem. Eng. J. 2021, 422, 130353.

[16] S. Y. Zhao , Y. Y. Zuo , T. Liu , S. Zhai , Y. W. Dai , Z. J. Guo , Y. Wang , Q. J. He , L. C. Xia , C. Y. Zhi , J. Bae , K. L. Wang , M. Ni , Adv. Energy Mater. 2021, 11, 2101749.

[17] M. Y. Ni , C. C. Ma , H. L. Huang , L. Han , X. B. Fu , Z. L. Yang , J. W. Li , L. K. Pan , M. Xu , ACS Appl. Energy Mater. 2022, 5, 6724.

[18] H. M. Yu , N. Rouelle , A. D. Qiu , J. A. Oh , D. M. Kempaiah , J. D. Whittle , M. Aakyiir , W. J. Xing , J. Ma , ACS Appl. Mater. Interfaces 2020, 12, 37977. 32697569 10.1021/acsami.0c05454

[19] Q. L. Ding , Z. X. Wu , K. Tao , Y. M. Wei , W. Y. Wang , B. R. Yang , X. Xie , J. Wu , Mater. Horiz. 2022, 9, 1356. 35156986 10.1039/d1mh01871j

[20] Q. F. Rong , W. W. Lei , L. Chen , Y. A. Yin , J. J. Zhou , M. J. Liu , Angew. Chem. Int. Ed. 2017, 56, 14159. 10.1002/anie.201708614 28940584

[21] M. Matsugami , T. Takamuku , T. Otomo , T. Yamaguchi , J. Phys. Chem. B 2006, 110, 12372. 16800561 10.1021/jp061456r

[22] H. L. Sun , Y. Zhao , S. L. Jiao , C. F. Wang , Y. P. Jia , K. Dai , G. Q. Zheng , C. T. Liu , P. B. Wan , C. Y. Shen , Adv. Funct. Mater. 2021, 31, 2101696.

[23] Y. Huang , M. Zhong , F. K. Shi , X. Y. Liu , Z. J. Tang , Y. K. Wang , Y. Huang , H. Q. Hou , X. M. Xie , C. Y. Zhi , Angew. Chem. Int. Ed. 2017, 56, 9141. 10.1002/anie.201705212 28631401

[24] H. L. Li , T. Lv , H. H. Sun , G. J. Qian , N. Li , Y. Yao , T. Chen , Nat. Commun. 2019, 10, 536. 30710074 10.1038/s41467-019-08320-z PMC6358613

[25] C. R. Chen , H. L. Qin , H. P. Cong , S. H. Yu , Adv. Mater. 2019, 31, 1900573. 10.1002/adma.201900573 30920707

[26] Q. F. Rong , W. W. Lei , J. Huang , M. J. Liu , Adv. Energy Mater. 2018, 8, 1801967.

[27] X. Jin , L. Song , H. Yang , C. Dai , Y. Xiao , X. Zhang , Y. Han , C. Bai , B. Lu , Q. J. Liu , Energy Environ. Sci. 2021, 14, 3075.

[28] H. W. Zhou , J. L. Lai , B. H. Zheng , X. L. Jin , G. X. Zhao , H. B. Liu , W. X. Chen , A. J. Ma , X. S. Li , Y. P. Wu , Adv. Funct. Mater. 2022, 32, 2108423.

[29] X. L. Li , D. Y. Lou , H. Y. Wang , X. Y. Sun , J. Li , Y. N. Liu , Adv. Funct. Mater. 2020, 30, 2007291.

[30] J. Huang , S. Peng , J. Gu , G. Chen , J. Gao , J. Zhang , L. Hou , X. Yang , X. Jiang , L. Guan , Mater. Horiz. 2020, 7, 2085.

[31] Q. C. Wang , Q. G. Feng , Y. P. Lei , S. H. Tang , L. Xu , Y. Xiong , G. Z. Fang , Y. C. Wang , P. Y. Yang , J. J. Liu , W. Liu , X. Xiong , Nat. Commun. 2022, 13, 3689. 35760794 10.1038/s41467-022-31383-4 PMC9237111

[32] H. Y. Zheng , R. H. Guan , Q. X. Liu , K. T. Ou , D. S. Li , J. Fang , Q. Fu , Y. Y. Sun , Electrochim. Acta 2022, 424, 140644.

[33] Y. Meng , L. F. Zhang , M. J. Peng , D. N. Shen , C. H. Zhu , S. Y. Qian , J. Liu , Y. F. Cao , C. L. Yan , J. Q. Zhou , T. Qian , Adv. Funct. Mater. 2022, 32, 2206653.

[34] Q. Zhang , X. L. Hou , X. Liu , X. Xie , L. J. Duan , W. Lu , G. H. Gao , Small 2021, 17, 2103091. 10.1002/smll.202103091 34643034

[35] Y. Guo , S. Dong , Anal. Chem. 1997, 69, 1904.

[36] Z. Zhang , J. C. Hao , Adv. Colloid Interface Sci. 2021, 292, 102408. 33932827 10.1016/j.cis.2021.102408

[37] C. Li , S. Feng , C. Li , Y. Sui , J. Shen , C. Huang , Y. Wu , W. Huang , Adv. Funct. Mater. 2020, 30, 2002163.

[38] A. Bhardwaj , J. Kaur , M. Wuest , F. Wuest , Nat. Commun. 2017, 8, 1. 28232747 10.1038/s41467-016-0009-6 PMC5431875

[39] S. Y. Zhuo , Z. G. Zhao , Z. X. Xie , Y. F. Hao , Y. C. Xu , T. Y. Zhao , H. J. Li , E. L. Knubben , L. Wen , L. Jiang , M. J. Liu , Sci. Adv. 2020, 6, eaax1464. 32064332 10.1126/sciadv.aax1464 PMC6994219

[40] Z. G. Zhao , K. J. Zhang , Y. X. Liu , J. J. Zhou , M. J. Liu , Adv. Mater. 2017, 29, 1701695.

[41] X. Su , H. Wang , Z. L. Tian , X. C. Duan , Z. H. Chai , Y. T. Feng , Y. X. Wang , Y. Fan , J. Y. Huang , ACS Appl. Mater. Interfaces 2020, 12, 29757. 32515578 10.1021/acsami.0c04933

[42] L. Fang , J. Zhang , W. Wang , Y. Zhang , F. Chen , J. Zhou , F. Chen , R. Li , X. Zhou , Z. Xie , ACS Appl. Mater. Interfaces 2020, 12, 56393. 33274913 10.1021/acsami.0c14472

[43] A. Bhardwaj , J. Kaur , M. Wuest , F. Wuest , Polymers 2020, 12, 2670.33198210

[44] E. Feng , J. Li , G. Zheng , Z. Yan , X. Li , W. Gao , X. Ma , Z. Yang , ACS Sustainable Chem. Eng. 2021, 9, 7267.

[45] F. Yu , P. Y. Yang , Z. Q. Yang , X. C. Zhang , J. Ma , Chem. Eng. J. 2021, 426, 131900.

[46] F. Chen , D. Zhou , J. H. Wang , T. Z. Li , X. H. Zhou , T. S. Gan , S. Handschuh-Wang , X. C. Zhou , Angew. Chem. Int. Ed. 2018, 57, 6568. 10.1002/anie.201803366 29656553

[47] J. C. Song , S. Chen , L. J. Sun , Y. F. Guo , L. Z. Zhang , S. L. Wang , H. X. Xuan , Q. B. Guan , Z. W. You , Adv. Mater. 2020, 32, 1906994. 10.1002/adma.201906994 31957099

[48] D. Zhou , F. Chen , J. H. Wang , T. Z. Li , B. J. Li , J. Zhang , X. H. Zhou , T. S. Gan , S. Handschuh-Wang , X. C. Zhou , J. Mater. Chem. B 2018, 6, 7366. 32254737 10.1039/c8tb02236d

[49] X. L. Li , H. Y. Wang , X. Y. Sun , J. Li , Y. N. Liu , ACS Appl. Energy Mater. 2021, 4, 12718.

[50] Z. H. Qin , D. Y. Dong , M. M. Yao , Q. Y. Yu , X. Sun , Q. Guo , H. T. Zhang , F. L. Yao , J. J. Li , ACS Appl. Mater. Interfaces 2019, 11, 21184. 31117467 10.1021/acsami.9b05652

[51] J. Wu , Z. H. Xu , Q. Wu , C. Liu , B. R. Yang , X. Gui , X. Xie , K. Tao , Y. Shen , Mater. Horiz. 2019, 6, 595.

[52] H. Sun , Y. Zhao , S. Jiao , C. Wang , Y. Jia , K. Dai , G. Zheng , C. Liu , P. Wan , C. Shen , Adv. Funct. Mater. 2021, 31, 2101696.

[53] J. Wu , Z. Wu , X. Lu , S. Han , B.-R. Yang , X. Gui , K. Tao , J. Miao , C. Liu , ACS Appl. Mater. Interfaces 2019, 11, 9405. 30763515 10.1021/acsami.8b20267

[54] Q. Y. Yu , Z. H. Qin , F. Ji , S. Chen , S. Y. Luo , M. M. Yao , X. J. Wu , W. W. Liu , X. Sun , H. T. Zhang , Y. L. Zhao , F. L. Yao , J. J. Li , Chem. Eng. J. 2021, 404, 126559.

[55] D. Y. Lou , C. S. Wang , Z. Y. He , X. Y. Sun , J. S. Luo , J. Li , Chem. Commun. 2019, 55, 8422. 10.1039/c9cc04239c 31257398

[56] C. R. Qin , A. Lu , Carbohydr. Polym. 2021, 274, 118667. 34702485 10.1016/j.carbpol.2021.118667

[57] H. Y. Wang , X. L. Li , D. Q. Jiang , S. Wu , W. L. Yi , X. Y. Sun , J. Li , J. Power Sources 2022, 528, 231210.

[58] J. Huang , J. Gu , J. Liu , J. Guo , H. Liu , K. Hou , X. Jiang , X. Yang , L. J. Guan , J. Mater. Chem. A 2021, 9, 16345.

[59] Z. He , W. Yuan , ACS Appl. Mater. Interfaces 2021, 13, 1474. 33393770 10.1021/acsami.0c18405

[60] X. L. Tong , Z. N. Tian , J. Y. Sun , V. C. Tung , R. B. Kaner , Y. L. Shao , Mater. Today 2021, 44, 78.

[61] D. Yu , X. Li , J. Xu , Sci. China Mater. 2019, 62, 1556.

[62] J. Lu , J. F. Gu , O. D. Hu , Y. H. Fu , D. Z. Ye , X. Zhang , Y. Zheng , L. X. Hou , H. Y. Liu , X. C. Jiang , J. Mater. Chem. A 2021, 9, 18406.

[63] X. T. Jin , L. Song , H. S. Yang , C. L. Dai , Y. K. Xiao , X. Q. Zhang , Y. Y. Han , C. C. Bai , B. Lu , Q. W. Liu , Y. Zhao , J. T. Zhang , Z. P. Zhang , L. T. Qu , Energy Environ. Sci. 2021, 14, 3075.

[64] W. C. Mai , Q. P. Yu , C. P. F. Y. Kang , B. H. Li , Adv. Funct. Mater. 2020, 30, 1909912.

[65] T. Cheng , Y. Z. Zhang , S. Wang , Y. L. Chen , S. Y. Gao , F. Wang , W. Y. Lai , W. J. Huang , Adv. Funct. Mater. 2021, 31, 2101303.

[66] F. Y. Hsieh , H. W. Han , X. R. Chen , C. S. Yang , Y. Wei , S. H. Hsu , Biomaterials 2018, 174, 31. 29777961 10.1016/j.biomaterials.2018.05.014

[67] X. Chen , M. A. Dam , K. Ono , A. Mal , H. Shen , S. R. Nutt , K. Sheran , F. Wudl , Science 2002, 295, 1698. 11872836 10.1126/science.1065879

[68] S. Talebian , M. Mehrali , N. Taebnia , C. P. Pennisi , F. B. Kadumudi , J. Foroughi , M. Hasany , M. Nikkhah , M. Akbari , G. Orive , Adv. Sci. 2019, 6, 1801664. 10.1002/advs.201801664 PMC6702654 31453048

[69] P. A. Pratama , M. Sharifi , A. M. Peterson , G. R. Palmese , ACS Appl. Mater. Interfaces 2013, 5, 12425. 24215583 10.1021/am403459e

[70] C. M. Madl , S. C. Heilshorn , Adv. Funct. Mater. 2018, 28, 1706046. 31558890 10.1002/adfm.201706046 PMC6761700

[71] Y. Chen , C. Y. Shi , Z. Y. Zhang , Q. Xu , H. Q. Hu , Y. Y. Wei , Polym. Bull. 2022, 79, 10723.

[72] C. E. Yuan , M. Q. Zhang , M. Z. Rong , J. Mater. Chem. A 2014, 2, 6558.

[73] X. Su , H. Wang , Z. Tian , X. Duan , Z. Chai , Y. Feng , Y. Wang , Y. Fan , J. Huang , ACS Appl. Mater. Interfaces 2020, 12, 29757. 32515578 10.1021/acsami.0c04933

[74] Y. Wei , L. Xiang , H. Ou , F. Li , Y. Zhang , Y. Qian , L. Hao , J. Diao , M. Zhang , P. Zhu , Adv. Funct. Mater. 2020, 30, 2005135.

[75] M. Chen , J. Chen , W. Zhou , X. Han , Y. Yao , C. P. Wong , Adv. Mater. 2021, 33, 2007559. 10.1002/adma.202007559 33511697

[76] J. Kamada , K. Koynov , C. Corten , A. Juhari , J. A. Yoon , M. W. Urban , A. C. Balazs , K. Matyjaszewski , Macromolecules 2010, 43, 4133.

[77] D. Chen , Y. Zhang , C. Ni , C. Ma , J. Yin , H. Bai , Y. Luo , F. Huang , T. Xie , Q. Zhao , Mater. Horiz. 2019, 6, 1013.

[78] B. J. Kim , D. X. Oh , S. Kim , J. H. Seo , D. S. Hwang , A. Masic , D. K. Han , H. J. Cha , Biomacromolecules 2014, 15, 1579. 24650082 10.1021/bm4017308

[79] T. Yamada , Y. Hayamizu , Y. Yamamoto , Y. Yomogida , A. Izadi-Najafabadi , D. N. Futaba , K. Hata , Nat. Nanotechnol. 2011, 6, 296. 21441912 10.1038/nnano.2011.36

[80] H. Yuk , T. Zhang , S. Lin , G. A. Parada , X. Zhao , Nat. Mater. 2016, 15, 190. 26552058 10.1038/nmat4463 PMC4762474

[81] F. N. Mo , G. J. Liang , Q. Q. Meng , Z. X. Liu , H. F. Li , J. Fan , C. Y. Zhi , Energy Environ. Sci. 2019, 12, 706.

[82] Z. Pei , L. Ding , C. Wang , Q. Meng , Z. Yuan , Z. Zhou , S. Zhao , Y. Chen , Energy Environ. Sci. 2021, 14, 4926.

[83] T. Xu , D. Z. Yang , S. Y. Zhang , T. Y. Zhao , M. Zhang , Z. Z. Yu , Carbon 2021, 171, 201.

[84] M. D. Galluzzo , D. M. Halat , W. S. Loo , S. A. Mullin , J. A. Reimer , N. P. Balsara , ACS Energy Lett. 2019, 4, 903.

[85] Y. Yang , K. P. Wang , Q. Zang , Q. Q. Shi , Y. W. Wang , Z. Y. Xiao , Q. Zhang , L. Wang , J. Mater. Chem. A 2022, 10, 11277.

[86] L. Yu , L. Yu , Q. Liu , T. Meng , S. Wang , X. L. Hu , Adv. Funct. Mater. 2022, 32, 2110653.

[87] Z. P. Huo , S. Y. Dai , C. G. Zhang , F. T. Kong , X. Q. Fang , L. Guo , W. Q. Liu , L. H. Hu , X. Pan , K. J. Wang , J. Phys. Chem. B 2008, 112, 12927. 18798664 10.1021/jp8052168

[88] Y. Wei , L. J. Xiang , P. H. Zhu , Y. Y. Qian , B. X. Zhao , G. Chen , Chem. Mater. 2021, 33, 8623.

[89] H. F. Wang , Y. J. Zhong , J. Q. Ning , Y. Hu , Chin. Chem. Lett. 2021, 32, 3733.

[90] W. Lu , Y. Yang , T. Y. Zhang , L. K. X. Ma , X. T. Luo , C. Q. Huang , J. Q. Ning , Y. J. Zhong , Y. Hu , J. Colloid Interface Sci. 2021, 590, 226. 33548606 10.1016/j.jcis.2021.01.050

[91] W. Lu , L. Yan , W. Q. Ye , J. Q. Ning , Y. J. Zhong , Y. Hu , J. Mater. Chem. A 2022, 10, 15267.

[92] W. Q. Ye , H. Y. Wang , J. Q. Ning , Y. J. Zhong , Y. Hu , J. Energy Chem. 2021, 57, 219.

[93] K. J. Sun , E. K. Feng , G. H. Zhao , H. Peng , G. G. Wei , Y. Y. Lv , G. F. Ma , ACS Sustainable Chem. Eng. 2019, 7, 165.

[94] Y. Guo , K. Q. Zheng , P. B. Wan , Small 2018, 14, 1704497. 10.1002/smll.201704497 29484807

[95] X. Lv , G. Li , D. Li , F. Huang , W. Liu , Q. Wei , J. Phys. Chem. Solids 2017, 110, 202.

[96] C. Meng , C. Liu , L. Chen , C. Hu , S. Fan , Nano Lett. 2010, 10, 4025. 20831255 10.1021/nl1019672

[97] G. Jung , H. Lee , H. Park , J. Kim , J. W. Kim , D. S. Kim , K. Keum , Y. H. Lee , J. S. Ha , Chem. Eng. J. 2022, 450, 138379.

[98] K. Hu , Z. P. Zhao , Y. Y. Wang , L. H. Yu , K. Liu , H. Wu , L. L. Huang , L. H. Chen , Y. H. Ni , J. Mater. Chem. A 2022, 10, 12092.

[99] S. K. Sami , S. Siddiqui , S. Shrivastava , N. E. Lee , Small 2017, 13, 1702142. 10.1002/smll.201702142 29045044

[100] H. Mu , X. Huang , W. Wang , X. Tian , Z. An , G. Wang , ACS Appl. Mater. Interfaces 2022, 14, 622. 34928149 10.1021/acsami.1c17186

[101] J. Huang , J. Gu , J. Liu , J. Guo , H. Liu , K. Hou , X. Jiang , X. Yang , L. Guan , J. Mater. Chem. A 2021, 9, 16345.

[102] W. Ge , S. Cao , Y. Yang , O. J. Rojas , X. Wang , Chem. Eng. J. 2021, 408, 127306.

[103] O. D. Hu , J. Lu , G. Q. Chen , K. Chen , J. F. Gu , S. Weng , L. X. Hou , X. Zhang , X. C. Jiang , ACS Sustainable Chem. Eng. 2021, 9, 9833.

[104] X. L. Hou , Q. Zhang , L. Y. Wang , G. H. Gao , W. Lu , ACS Appl. Mater. Interfaces 2021, 13, 12432. 33657789 10.1021/acsami.0c18741

[105] J. W. Gu , Z. Yuan , H. Y. Wang , J. L. Shen , J. Q. Ning , Y. J. Zhong , Y. Hu , Chem. Eng. J. 2022, 448, 137711.

[106] Y. Yang , D. L. Chen , H. Y. Wang , P. C. Ye , Z. T. Ping , J. Q. Ning , Y. J. Zhong , Y. Hu , Chem. Eng. J. 2022, 431, 133250.

[107] F. W. Ming , Y. P. Zhu , G. Huang , A. H. Emwas , H. F. Liang , Y. Cui , H. N. Alshareef , J. Am. Chem. Soc. 2022, 144, 7160. 35436108 10.1021/jacs.1c12764

[108] W. Sun , F. Wang , B. Zhang , M. Y. Zhang , V. Kupers , X. Ji , C. Theile , P. Bieker , K. Xu , C. S. Wang , M. Winter , Science 2021, 371, 46. 33384369 10.1126/science.abb9554

[109] T. Chen , Y. A. Wang , Y. Yang , F. Huang , M. K. Zhu , B. T. W. Ang , J. M. Xue , Adv. Funct. Mater. 2021, 31, 2101607.

[110] M. Chen , W. Zhou , A. Wang , A. Huang , J. Chen , J. Xu , C. P. Wong , J. Mater. Chem. A 2020, 8, 6828.

[111] Y. Liu , H. He , A. Gao , J. Ling , F. Yi , J. Hao , Q. Li , D. Shu , Chem. Eng. J. 2022, 446, 137021.

[112] S. Guo , L. P. Qin , C. Hu , L. Y. Li , Z. G. Luo , G. Z. Fang , S. Q. Liang , Adv. Energy Mater. 2022, 12, 2200730.

[113] T. T. Wei , Y. K. Ren , Z. Q. Li , X. X. Zhang , D. H. Ji , L. H. Hu , Chem. Eng. J. 2022, 434, 134646.

[114] Q. Liu , R. P. Chen , L. Xu , Y. Liu , Y. H. Dai , M. Huang , L. Q. Mai , ACS Energy Lett. 2022, 7, 2825.

[115] J. W. Wang , Y. Huang , B. B. Liu , Z. X. Li , J. Y. Zhang , G. S. Yang , P. Hiralal , S. Y. Jin , H. Zhou , Energy Storage Mater. 2021, 41, 599.

[116] F. N. Mo , G. J. Liang , D. H. Wang , Z. J. Tang , H. F. Li , C. Y. Zhi , Ecomat 2019, 1, e12008.

[117] Z. Zhao , Z. K. Yuan , Z. S. Fang , J. H. Jian , J. Li , M. J. Yang , C. S. Mo , Y. Zhang , X. H. Hu , P. Li , S. Y. Wang , W. Hong , Z. K. Zheng , G. F. Ouyang , X. D. Chen , D. S. Yu , Adv. Sci. 2020, 7, 2001501. 10.1002/advs.202001501 PMC7284209 32537422

[118] Y. Rao , S. Chen , Q. Yue , Y. J. Kang , ACS Catal. 2021, 11, 8097.

[119] X. X. Yan , Y. Ha , R. B. Wu , Small Methods 2021, 5, 2000827. 10.1002/smtd.202000827 34927848

[120] J. Park , M. Park , G. Nam , J. S. Lee , J. Cho , Adv. Mater. 2015, 27, 1396. 25532853 10.1002/adma.201404639

[121] Y. Y. Zuo , K. L. Wang , M. H. Wei , P. F. Zhang , S. Y. Zhao , P. C. Pei , H. W. Wang , Z. Chen , N. Shang , Chem. Eng. J. 2023, 452, 139301.

[122] K. Tang , C. Yuan , Y. Xiong , H. Hu , M. Wu , Appl. Catal., B 2020, 260, 118209.

[123] L. Yan , Z. Y. Xu , W. K. Hu , J. Q. Ning , Y. J. Zhong , Y. Hu , Nano Energy 2021, 82, 105710.

[124] Y. A. Zhang , D. S. Wu , F. L. Huang , Y. B. Cai , Y. G. Li , H. Z. Ke , P. F. Lv , Q. F. Wei , Adv. Funct. Mater. 2022, 32, 2203204.

[125] M. Xu , D. G. Ivey , W. Qu , Z. Xie , J. Power Sources 2014, 252, 327.

[126] Q. Nian , J. Wang , S. Liu , T. Sun , S. Zheng , Y. Zhang , Z. Tao , J. Chen , Angew. Chem. Int. Ed. 2019, 58, 16994. 10.1002/anie.201908913 31541502

[127] D. Q. Jiang , H. Y. Wang , S. Wu , X. Y. Sun , J. Li , Small Methods 2022, 6, 2101043. 10.1002/smtd.202101043 35041284

[128] R. Chen , X. Xu , S. Peng , J. Chen , D. Yu , C. Xiao , Y. Li , Y. Chen , X. Hu , M. Liu , ACS Sustainable Chem. Eng. 2020, 8, 11501.

[129] X. Chen , Z. R. Hou , G. X. Li , W. Yu , Y. Xue , G. Y. Niu , M. Y. Xin , L. Yang , C. Z. Meng , S. J. Guo , Nano Energy 2022, 101, 107606.

[130] X. Zhao , J. Sun , J. Ma , T. Liu , Z. Guo , Z. Yang , W. Yao , X. Jiang , Sustainable Mater. Technol. 2022, 32, e00420.

[131] J. X. Gong , C. C. L. Schuurmans , A. M. van Genderen , X. Cao , W. L. Li , F. Cheng , J. J. He , A. Lopez , V. Huerta , J. Manriquez , R. Q. Li , H. B. Li , C. Delavaux , S. Sebastian , P. E. Capendale , H. M. Wang , J. W. Xie , M. F. Yu , R. Masereeuw , T. Vermonden , Y. S. Zhang , Nat. Commun. 2020, 11, 1267. 32152307 10.1038/s41467-020-14997-4 PMC7062888

[132] T. Xu , K. Liu , N. Sheng , M. H. Zhang , W. Liu , H. Y. Liu , L. Dai , X. Y. Zhang , C. L. Si , H. S. Du , K. Zhang , Energy Storage Mater. 2022, 48, 244.

[133] Z. Z. Zhang , Y. Li , R. Xu , W. Zhou , Y. Li , S. T. Oyakhire , Y. Wu , J. Xu , H. Wang , Z. Yu , Science 2022, 375, 66. 34990230 10.1126/science.abi8703

